# Electrostatic-modulated interfacial polymerization toward ultra-permselective nanofiltration membranes

**DOI:** 10.1016/j.isci.2021.102369

**Published:** 2021-03-26

**Authors:** Xinda You, Ke Xiao, Hong Wu, Yafei Li, Runlai Li, Jinqiu Yuan, Runnan Zhang, Zhiming Zhang, Xu Liang, Jianliang Shen, Zhongyi Jiang

**Affiliations:** 1 Key Laboratory for Green Chemical Technology of Ministry of Education, School of Chemical Engineering and Technology, Tianjin University, Tianjin 300072, China; 2 Collaborative Innovation Center of Chemical Science and Engineering (Tianjin), Tianjin 300072, China; 3 Tianjin Key Laboratory of Membrane Science and Desalination Technology, Tianjin University, Tianjin 300072, China; 4 Joint School of National University of Singapore and Tianjin University, International Campus of Tianjin University, Binhai New City, Fuzhou 350207, China; 5 Chemistry and Chemical Engineering Guangdong Laboratory, Shantou 515031, China; 6 Department of Chemistry, National University of Singapore, Singapore 117549, Singapore

**Keywords:** Supramolecular Materials, Materials Science, Materials Chemistry, Materials Synthesis, Polymers

## Abstract

Interfacial polymerization (IP) is a platform technology for ultrathin membranes. However, most efforts in regulating the IP process have been focused on short-range H-bond interaction, often leading to low-permselective membranes. Herein, we report an electrostatic-modulated interfacial polymerization (eIP) *via* supercharged phosphate-rich substrates toward ultra-permselective polyamide membranes. Phytate, a natural strongly charged organophosphate, confers high-density long-range electrostatic attraction to aqueous monomers and affords tunable charge density by flexible metal-organophosphate coordination. The electrostatic attraction spatially enriches amine monomers and temporally decelerates their diffusion into organic phase to be polymerized with acyl chloride monomers, triggering membrane sealing and inhibiting membrane growth, thus generating polyamide membranes with reduced thickness and enhanced cross-linking. The optimized nearly 10-nm-thick and highly cross-linked polyamide membrane displays superior water permeance and ionic selectivity. This eIP approach is applicable to the majority of conventional IP processes and can be extended to fabricate a variety of advanced membranes from polymers, supermolecules, and organic framework materials.

## Highlights

• Electrostatic-modulated interfacial polymerization is proposed for the first time
• Electrostatic attraction regulates the spatial-temporal distribution of amine monomers
• Monomer regulation leads to reduced thickness and enhanced cross-linking of membrane
• Ultrathin and highly cross-linked polyamide membrane displays superior permselectivity

## Graphical abstract

**Figure:**
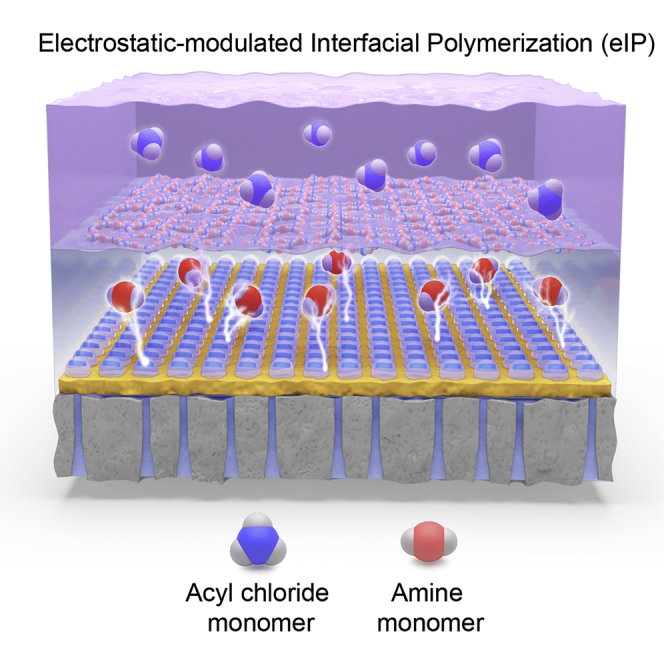

## Introduction

Interfacial polymerization (IP) is a platform technology for fabricating ultrathin membranes by confining chemical reactions at the immiscible biphasic interface (Wang et al., 2020). The combination of technological universality and process designability makes IP attractive for a great number of advanced membrane materials, including polymers (Jiang et al., 2018; Tan et al., 2018), supermolecules (Qin et al., 2017), metal-organic frameworks (MOFs) (Brown et al., 2014; Makiura et al., 2010), and covalent organic frameworks (COFs) (Dey et al., 2017; Hao et al., 2018; Khan et al., 2020). Thereinto, the manufacturing of polyamide nanofiltration (NF) membranes represents the large-scale application of IP technology, providing an energy-efficient and sustainable paradigm for water desalination, water softening, and ionic separation (Peng et al., 2020; Werber et al., 2016). For a typical IP process, the porous ultrafiltration (UF) membrane is successively immersed in aqueous phase with amine monomer (piperazine, PIP) and organic phase with acyl chloride monomer (trimesoyl chloride, TMC) to form a polyamide membrane spanning tens to hundreds of nanometers thick (You et al., 2017). Ever thinner synthetic membrane approaching an 8-nm-thick cell membrane with short mass transport pathway can harvest high permeability (Hao et al., 2018; Jiang et al., 2018; Karan et al., 2015; Li et al., 2020; Yuan et al., 2019), and sufficient cross-linking is essential for generating high permselectivity (Liang et al., 2020). However, the too fast polymerization reaction rate of the IP process hampers the precise structural manipulation of polyamide membranes with 10-nm-scale thickness and high cross-linking degree (Freger, 2003).

Spatial-temporal distribution of aqueous monomer governs the IP process and reflects as monomer concentration and monomer diffusion rate, respectively (Freger, 2003). Currently, most efforts for monomer regulation are focused on short-range H-bond interactions (Guan et al., 2020; Karan et al., 2015; Li et al., 2020; Tan et al., 2018; Wang et al., 2017; Wu et al., 2019; You et al., 2017; Yuan et al., 2019). For temporal regulation, retarded amine monomer diffusion across the interface and slowed down membrane formation has been realized by introducing H-bond generating materials into the aqueous phase to closely interact with the amine monomer (Li et al., 2020; Tan et al., 2018). This diffusion-based strategy for temporal monomer distribution yields thinner membranes but usually lacks spatial monomer regulation for sufficient cross-linking and, in many cases, damages membrane integrity and selectivity (Guan et al., 2020; Wang et al., 2017; You et al., 2017). As an alternative, spatial regulation by enriching aqueous monomers at the interface increases the interfacial monomer concentration and triggers the rapid formation of a dense primary layer. This dense primary layer could hinder amine monomer diffusion and inhibit further membrane growth, namely the self-sealing process. By depositing H-bond generating materials on substrate surface to adsorb aqueous monomers and increase monomer concentration, nearly 10-nm-thick polyamide membranes have been achieved by promoted self-sealing (Karan et al., 2015; Wu et al., 2019; Yuan et al., 2019). Nonetheless, the concentration effect based on H-bond interaction is spatially restricted in terms of action range (ca. 1–3 nm) (Hakala et al., 2006) and geometrical distribution (directionality and saturability) (Modig et al., 2003). Insufficient monomer enrichment usually forms a loose polyamide membrane with a relatively low cross-linking degree (ca. 50%–60%) and thus only moderate salt rejection (<95%, Na_2_SO_4_) (Wang et al., 2018; Wu et al., 2019; Yuan et al., 2019). Besides, this spatial limitation makes these substrates unable to regulate the temporal distribution of monomer given that the distance between substrate and interface exceeds the range of H-bond interaction. Developing an innovative modulating strategy of IP process based on long-range interaction may create a promising opportunity for fabricating ultrathin and highly cross-linked polyamide membranes affording high permselectivity.

Electrostatic interaction is a ubiquitous long-range interaction in aqueous media (Bowen and Sharif, 1998; Zhang et al., 2019) because most dissolved solutes can be charged through dissociation or adsorption. The biological system illustrates the superiority of electrostatic adsorption by forming fouling-resistant hydration shells around cell membranes from electrostatically absorbed water molecules with charged phosphatidylcholine head groups (Chen et al., 2005; He et al., 2016; Jiang and Cao, 2010). This electrostatic-induced hydration by charged groups could adsorb seven times more water molecules than the H-bond-induced hydration by traditional hydrophilic materials (*e.g.*, polyethylene glycol) due to the longer interaction range and higher interaction density of electrostatic interaction (Wu et al., 2012). Thereby, the general electrostatic interaction in the aqueous phase may influence charged monomers in a broader range and thus manipulate their distribution during the IP process.

**Figure 1:**
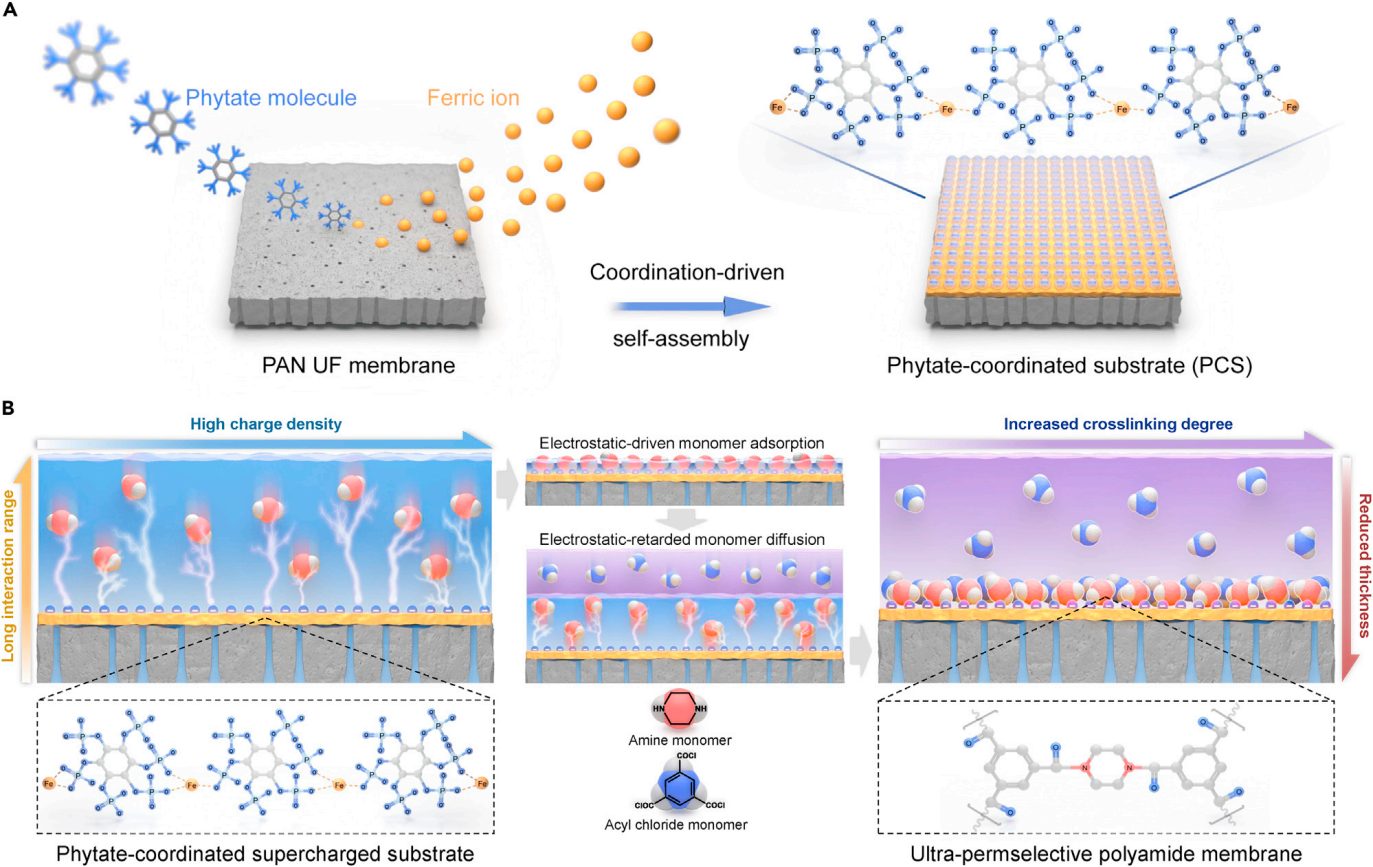
Schematic procedure of electrostatic-modulated interfacial polymerization (eIP) (A) Coordination-driven self-assembly of Fe^3+^-phytate complex on PAN UF membrane for phytate-coordinated substrate (PCS). (B) Interfacial polymerization of ultrathin and highly cross-linked polyamide membrane with superior permselectivity.

Herein, we propose an electrostatic-modulated interfacial polymerization (eIP) on a supercharged phosphate-rich substrate to achieve ultra-permselective polyamide membranes (Figure 1). Phytate is a natural organophosphate rich in phosphate groups featuring an ultrahigh theoretical spatial charge density (~6.735 × 10^2^°C nm^−3^) (Song et al., 2016), and the strong electron-donating phosphate group enables coordination-driven self-assembly with metal electron acceptor to anchor phytate on various substrates (Li et al., 2016; You et al., 2019; Yu et al., 2020). In this work, phytate molecules were anchored onto porous UF membrane by coordination-driven self-assembly to form a phytate-coordinated substrate (PCS), affording strong electrostatic interaction with aqueous monomers (Figure 1A). The electrostatic interaction between PCS and amine monomer is precisely regulated by the flexible metal-organophosphate coordination that generates tunable surface charge density, thus modulating the monomer adsorption and diffusion. The electrostatic attraction spatially enriches amine monomers and temporally retards their diffusion into organic phase to polymerize with acyl chloride monomer, triggering the formation of ultrathin defect-free polyamide membranes with tunable thickness and high cross-linking degree (Figure 1B). The optimized ultrathin and highly cross-linked polyamide membrane displays superior permselectivity for nanofiltration. This work demonstrates that strong electrostatic interaction could offer a generic strategy to accelerate IP technology development toward high-performance ultrathin membranes.

## Results

### Electrostatic-regulated monomer adsorption and diffusion

**Figure 2:**
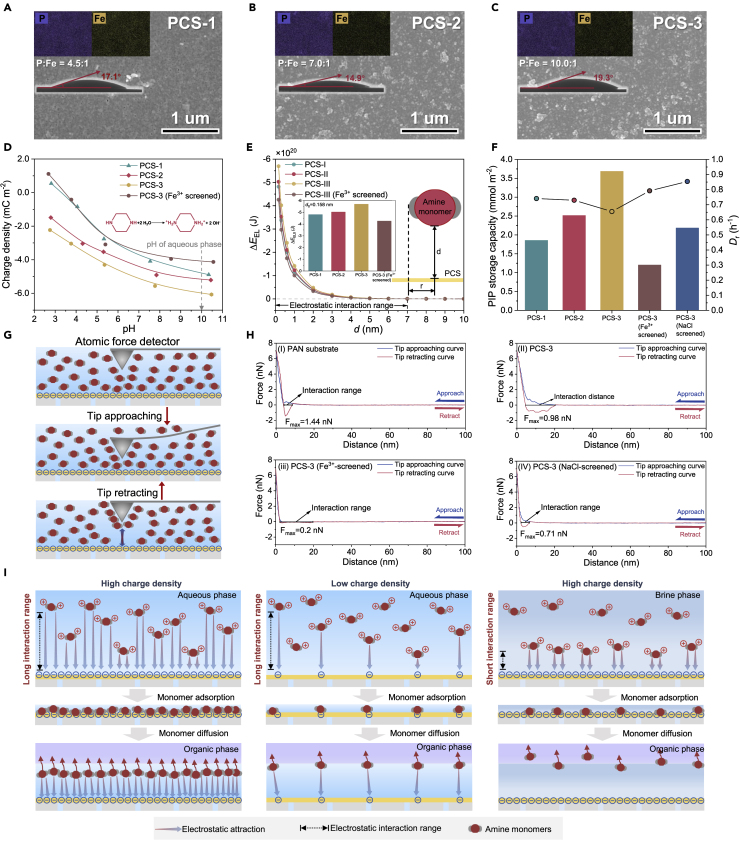
Electrostatic-regulated monomer adsorption and diffusion (A−C) Morphology of PCS with varied anchoring contents of phytate. Insets: Elemental distribution (top) and water contact angles (bottom) of PCS. (D) pH-dependent surface chargeability of PCS. Inset: Protonation equation of PIP in the aqueous phase. (E) Electrostatic interaction energy (Δ*E*_EL_, J) between protonated amine monomer and charged PCS. Inset: Physical model of XDLVO theory for calculating interaction energy and interaction energy at minimum equilibrium distance (Δ*E*_EL0_, *d* = 0.158 nm) of PCS. (F) Piperazine storage capacity and relative monomer diffusion rate of PCS. (G) Schematic diagrams of interaction measurement between PIP and substrate by an atomic force detector. (H) Force-distance curves of the wetting substrate surfaces. The atomic-scale PIP-monomer interaction of PAN (i), PCS-3 (ii), PCS-3 (Fe^3+^-screened) (iii), and PCS-3 (NaCl-screened) (iv) are measured. (I) Schematic diagrams of electrostatic-regulated monomer adsorption and diffusion under different charge density and interaction range.

We directly anchored phytate molecules onto polyacrylonitrile (PAN) UF membranes by coordination-driven self-assembly with Fe^3+^ ions as an electron acceptor to form a robust P-O-Fe bond (Figure S1) (You et al., 2019; Yu et al., 2020). As shown in Figures 2A−2C, the assembled phytate-Fe^3+^ network forms a coating layer on the porous PAN membrane surface to give a PCS. The Fourier transform infrared (FTIR) spectroscopy characteristic peak at 1066 cm^−1^ corresponding to P-O-C manifests the successful anchoring of -PO_4_^2−^ groups on PAN (Figure S2), and the mapping images of phosphorus element on the surface of PCS indicate the uniform distribution of -PO_4_^2−^ groups (insets in Figures 2A−2C).

One exotic characteristic of metal-phytate coordination is structural tunability depending on the ratio between ligand and metal ion (You et al., 2019). By regulating the addition amount of phytate in assembly solution, we fabricated three typical PCSs with varied P:Fe elemental ratios of 4.5:1, 7.0:1, and 10.0:1 and denoted them as PCS-1, PCS-2, and PCS-3, respectively (Figures 2A−2C). Besides, by further immersing PCS-3 in Fe^3+^ solution, the phosphate groups can be substantially screened by the coordination with Fe^3+^, displaying a P:Fe elemental ratio of 2.7:1 (Figure S3). Notably, the small water contact angles (15°–23°) and fast spreading of water drop on the surface indicate that these PCSs bear desirable hydrophilicity benefited from the high hydration energy of the phosphate group (Figure S4) (You et al., 2019). This high hydrophilicity is advantageous for sufficient contact between the substrate and the aqueous phase to guarantee the effectiveness of electrostatic interaction between PIP and PCS.

We quantitively evaluated the surface charge density of PCS according to the Gouy-Chapman equation by measuring the zeta potential (Bowen and Cao, 1998). As shown in Figure 2D, with the increase of pH, PCS becomes more negatively charged due to the deprotonation of phosphate groups under the alkaline environment. Since PIP protonation offers a hydroxyl-rich environment (pH = 10.0, inset in Figure 2D), the PCS would also be negatively charged when immersed in the PIP-containing solution. Consequently, the charge density of PCS under pH = 10.0 may reflect their chargeability of attracting positively charged PIP molecules in the aqueous phase. As envisaged, regulating the anchored phytate content creates a tunable charge density of −4.84 mC m^−2^, −5.15 mC m^−2^, and −6.02 mC m^−2^ for PCS-1, PCS-2, and PCS-3, respectively, which is around 2–3 times higher than that of traditional polyelectrolytes (Zhang et al., 2019).

Additionally, the Fe^3+^-screened PCS-3 exhibits a weakened charge density of −4.09 mC m^−2^, manifesting the critical contribution of the phosphate group in generating a negative charge. To further quantify the electrostatic interaction between PIP and PCS, we calculated the distance-dependent electrostatic interaction energy (Δ*E*_EL_) of the PIP-PCS binary system based on the extended Derjaguin-Landau-Verwey-Overbeek (XDLVO) theory (Figure 2E) (Liu and Zhao, 2005). According to the second law of thermodynamics, Δ*E*_EL_<0 indicates the spontaneity of electrostatic-driven PIP adsorption on PCS. Consequently, the electrostatic interaction range of PCS toward PIP can be calculated to be ~7 nm by setting the Δ*E*_EL_ = 0 J, embodying the superior long-range character of electrostatic interaction than traditional H-bond interaction (~1–3 nm) (Hakala et al., 2006; Rozenberg et al., 2000). In accordance with the variation trend of charge density, the Δ*E*_EL_ decreases with the enhanced charged density, hinting the higher possibility to trigger PIP adsorption. When setting distance as minimum equilibrium distance (*d*_0_ = 0.158 nm), the Δ*E*_EL0_ was determined to be −4.82 × 10^−20^ J, −5.03 × 10^−20^ J, −5.70 × 10^−20^ J, and −4.26 × 10^−20^ J for PCS-1, PCS-2, PCS-3, and Fe^3+^-screened PCS-3, respectively. The above results indicate the successful manipulation of electrostatic interaction between PCS and PIP by tuning the surface charge density.

Adsorption and diffusion experiments were performed to validate the electrostatic monomer regulation by PCS. To simulate the IP procedure, we first immersed the PCS into PIP solution followed by air drying and subsequent immersion in the organic phase (*n*-heptane). By detecting PIP concentration in the organic phase, the PIP storage capacity of substrates was obtained based on the diffusion kinetics of PIP (Figures 2F, S5, and S6). With the increased Δ*E*_EL_, the PIP storage capacity turns to be 1.86 mmol m^−2^, 2.52 mmol m^−2^, and 3.68 mmol m^−2^ for PCS-1, PCS-2, and PCS-3, respectively, around 35.8%, 83.2%, and 168.6% higher than that of pristine PAN membrane (1.37 mmol m^−2^). By measuring the porosity of PCS, we found that all PCSs show an extremely low Brunauer-Emmett-Teller (BET) value around 1.49–2.10 m^2^ g^−1^ that is two orders of magnitude smaller than that of the previously reported porous material for monomer adsorption (Yuan et al., 2019), indicating that the monomer adsorption in this work is not caused by nanopores in the phytate-Fe^3+^ network (Figure S7).

To verify that the electrostatic interaction dominates the PIP adsorption, we screened the electrostatic-driven adsorption of PCS-3 in horizontal and vertical dimensions. As mentioned above, further coordination of PCS-3 by Fe^3+^ gives rise to an exceedingly shadowed charge density of −4.09 mC m^−2^ (decreased by 32.1%) and Δ*E*_EL0_ of −4.26 × 10^−20^ J (decreased by 25.3%). Accordingly, the PIP storage capacity of Fe^3+^-screened PCS-3 is also weakened down to 1.20 mmol m^−2^ (decreased by 67.4%), indicating that high surface charge density contributes to the enhanced PIP adsorption (Figures 2E and S6A). The long-range character of electrostatic interaction was further investigated by introducing electrolytes (5000 ppm of NaCl) into aqueous solution during PIP adsorption. The electrostatic interaction range is strongly dependent on the ion strength of the aqueous solution, which can be quantified by Debye length *λ*_D_ as follows (Yan et al., 2017):

(Equation 1) $$\lambda_{D}=\frac{3.04\times 10^{-10}}{z_{i}\sqrt{c_{i}}}$$

where *z*_i_ and *c*_i_ are the valency and concentration of ion i, respectively. For the brine phase containing 5000 ppm of NaCl, the Debye length is calculated to be 1.03 nm (denoted as NaCl-screened PCS-3), indicating the electrostatic interaction range in this environment is reduced from ~7 nm to ~1 nm. The considerably shortened interaction range gives rise to a 40.8% reduction of PIP adsorption (2.18 mmol m^−2^, Figures 2E and S6B).

Apart from the monomer enrichment to influence spatial distribution, the monomer diffusion kinetics also influences temporal monomer distribution and the IP process (Li et al., 2020). For the porous PAN membrane, the PIP is stored in the nanopore by capillary effect (Yang et al., 2017). These nanopores were covered by a metal-phytate layer, enabling on-surface monomer adsorption (Figures 2A−2C). The in-pore monomer storage of the porous PAN leads to a longer distance for PIP molecule diffusion into the organic phase than PCS featuring on-surface monomer storage. Thereby, the relative monomer diffusion rate (*D*_r_) of PAN (0.509 hr^−1^) is smaller than that of PCS (Figure S8 and Table S1). Besides, the enhanced electrostatic attraction may retard PIP diffusion and decrease *D*_r_ (PCS-1: 0.741 hr^−1^, PCS-2: 0.730 hr^−1^, PCS-3: 0.655 hr^−1^). This electrostatic-retarded PIP diffusion is also confirmed by the increased *D*_r_ after screening electrostatic interaction (Fe^3+^-screened PCS-3: 0.792 hr^−1^, NaCl-screened PCS-3: 0.854 hr^−1^), indicating that the charge density and interaction range jointly influenced the monomer diffusion.

To measure underwater atomic-scale interaction between PIP monomer and the substrate surface, we utilized the state-of-the-art atomic force detecting technology with the tip from an atomic force microscope (AFM) (Shen et al., 2020). As schematized by Figure 2G, the detector tip of the AFM was immersed in the PIP solution, during which the PIP monomer would be adsorbed by the sharp tips. When measurement begins, the PIP-adsorbed tip approaches the substrate to build up interaction. Afterward, the PIP-adsorbed tip retracts away from the substrate surface and suffered from the drag caused by PIP-substrate interaction. This atomic-scale PIP-substrate interaction force could be quantified by force-distance curves (Figure 2H). As shown in Figure 2H, the PAN features linear force-distance relation [Figure 2H(i)] while three kinds of PCS-3 display nonlinear force-distance relation [Figure 2H(ii-iv)]. These divergent force-distance relations indicate the different force types, where a typical electrostatic force is directly proportional to *d*^−2^. More importantly, the PCS-3 exhibits a three times broader interaction range with PIP than PAN, demonstrating the long-range character of electrostatic force. After screening the charge density [Figure 2H(iii)] and interaction range [(Figure 2H(iv)] of PCS-3, the force intensity (F_max_) and interaction range between PCS-3 and PIP were substantially weakened, respectively, in agreement with the XDLVO theory and Debye electrostatic screening theory, and also explained the unique electrostatic-modulated spatial-temporal distribution of PIP.

As illustrated in Figure 2I, the high density and long action range of electrostatic interaction from PCS synergistically contribute to the spatial enrichment and temporal retardation of PIP monomers. When immersed in the aqueous phase, the electrostatic attraction may adsorb PIP on the surface, while during monomer diffusion, the electrostatic attraction may slow down monomer transport across the interface. The increased monomer concentration and retarded monomer diffusion are both advantageous for reducing membrane thickness. Moreover, the assembly process can be easily scaled up (Figure S9).

### Electrostatic-modulated interfacial polymerization

**Figure 3:**
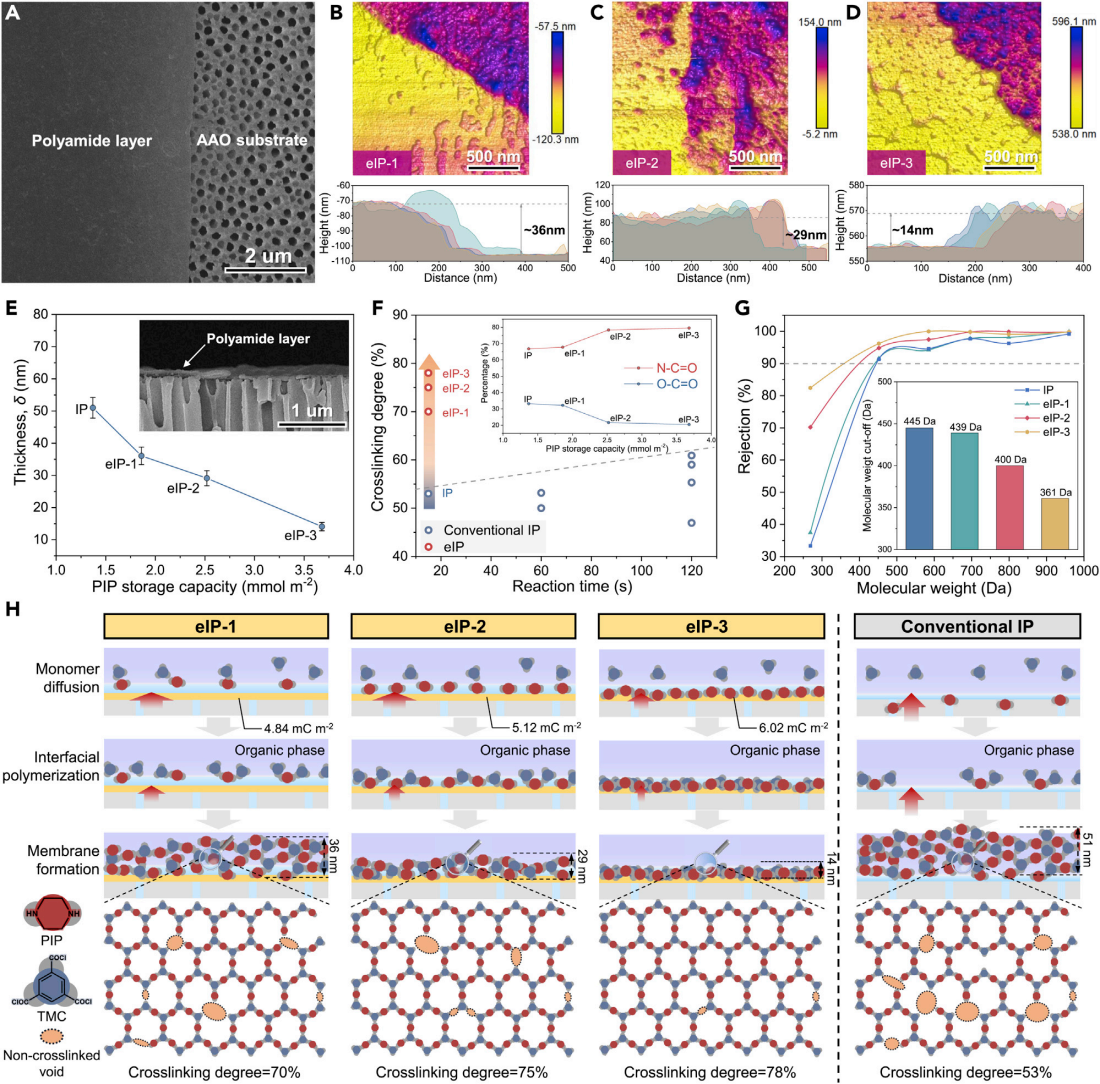
Electrostatic-modulated interfacial polymerization (A) Surface morphology of the polyamide membrane transferred onto the AAO substrate. (B−D) AFM images and corresponding height profiles of substrate-free eIP polyamide membranes. (E) Thicknesses of polyamide membranes prepared by conventional IP and eIP. Inset: Cross-sectional morphology of ultrathin polyamide membranes on the AAO substrate. (F) Summary of cross-linking degree of polyamide membranes with different reaction times. Inset: Chemical group composition of polyamide membranes influenced by PIP storage capacity. (G) Molecular weight cutoff (MWCO) of polyamide membranes. (H) Schematic diagrams of mechanism for eIP with varied surface charge density.

By immersing PIP-adsorbed substrates into the organic phase containing TMC monomers in 15 s, the PIP monomers diffuse into the organic phase to react with TMC and form a polyamide membrane (Table S2). The FTIR peaks at 1622 cm^−1^ and 1450 cm^−1^ corresponding to C=O and C-N, respectively, reveal the successful formation of polyamide on both the PAN UF membrane and PCS (Figure S10). To characterize the thickness of the polyamide layer, we immersed the composite membrane in dimethyl formamide and washed it with ethanol to obtain substrate-free polyamide membranes (Figure S11). As shown in Figure 3A, the polyamide membrane transferred onto an anodized aluminum oxide (AAO) substrate displays an intact structure, validating the possibility to measure membrane thickness by an AFM. According to the height profiles from AFM images, the thicknesses of eIP-1, eIP-2, and eIP-3 are ~36 nm, ~29 nm, and ~14 nm, respectively (Figures 3B−3D), much lower than those of the polyamide membrane prepared by the conventional IP (~51 nm, Figures 3E and S12). The PIP storage capacity (mmol m^−2^) of the substrate directly influences the PIP concentration for interfacial reaction and thus the thickness (*δ*, nm) of the resulting polyamide membrane according to the following relations (Freger, 2003):

(Equation 2) $$\delta ∼{\left[\frac{\mathrm{LD}}{k(C_{a}f_{a}+C_{o}f_{o})}\right]}^{1/3}$$

where *C*_*a*_ is the PIP concentration at the organic side near the interface, *C*_*o*_ is the TMC concentration in the organic phase, *L* is the thickness of the diffusion boundary layer at the interface, *D* is the diffusivity of PIP in the organic phase, *k* is the reaction rate constant between PIP and TMC, and *f* is the functionality of monomers. The enhanced PIP storage may increase the *C*_*a*_ of the organic phase, as demonstrated by the higher PIP concentration with the same diffusion time (Figure S5). Hence, the polyamide layer thickness decreases from 51.0 ± 3.2 nm down to 14.1 ± 1.3 nm along with the increased PIP storage capacity from 1.37 mmol m^−2^ to 3.68 mmol m^−2^ (Figure 3E). As discussed in section 2.2, the PIP monomer diffusion from PAN is slower than that of PCS, validating that the higher monomer concentration is responsible for the thinner polyamide layer on eIP membranes than the IP membrane. As for eIP membranes, the electrostatic-retarded monomer diffusion also contributes to the decreased polyamide layer thickness (Figure 2F). For instance, the eIP-3 membrane displays a thinner structure (~14.1 nm) than that predicted by Freger's model (~25.5 nm), manifesting the influence of retarded monomer diffusion by supercharged PCS-3 (Figure S13).

The cross-linking degree of the resultant polyamide membrane reflects the efficiency of the interfacial reaction and is crucial for membrane selectivity. As shown in Figure 3F, the cross-linking degrees of eIP-1, eIP-2, and eIP-3 membranes are 70%, 75%, and 78%, respectively, much higher than 53% of the IP membrane (Figure S14). This enhanced cross-linking increases the N-C=O content from 66.8% to 79.6% and decreases the O-C=O content from 33.2% to 20.4% (inset in Figure 3F), indicating that sufficient PIP enrichment on the substrate surface increases the *C*_a_, accelerates the cross-linking between biphasic monomers, and promotes the membrane formation process. The decreased molecular weight cutoff (MWCO) from 445 Da to 361 Da further confirms the denser structure of polyamide membranes (inset in Figures 3G and S15). When compared with previously reported polyamide membranes by conventional IP (cross-linking degree of 40%–60%, reaction time of 60–120 s), the polyamide membrane fabricated by eIP yields substantially higher cross-linking degree (70%–80% in 15 s) in 3–7 times shorter time (Figures 3F and Table S3). By conventional IP, the MWCO of the resultant polyamide membrane can reach the same level of the eIP-2 membrane when prolonging the reaction time by three times (Figure S16). These results demonstrate that eIP technology could allow ultrafast and controllable fabrication of ultrathin dense polyamide membranes.

The elucidation of the eIP process as illustrated in Figure 3H would provide a basis to better understand how the PCS influences membrane formation. First of all, three PCSs feature desirable hydrophilicity for the uniform spreading and good contact with the aqueous phase. Second, the supercharged PCS offers high-density long-range electrostatic interaction to attract a wide range of PIP monomers and enriches them on the substrate surface. Upon contacting the organic phase, the uniformly enriched PIP monomers diffuse across the aqueous-organic interface and undergo a cross-linking reaction with the TMC monomer. The PCS bearing high charge density induces monomer enrichment and slows down monomer diffusion by electrostatic attraction, thus generating a denser primary layer with a higher cross-linking degree. This dense primary layer hinders subsequent PIP diffusion and interfacial reaction between monomers, promoting self-sealing and forming thinner polyamide membranes. This mechanism is further supported by the increased thickness of Fe^3+^-screened and NaCl-screened eIP-3 membranes (Figure S17). Besides, we also observed diverse nanostructure on the surface of eIP membranes (Figures S18−S20), which may be attributed to the aqueous template effect on the hydrophilic substrate (Jiang et al., 2019, 2020). These nanostructures of the eIP membranes only induce a very limited increasement of surface area in the range of 2%–9% (Figure S21), exerting insignificant influence on membrane performance.

### Permselectivity of polyamide membranes

**Figure 4:**
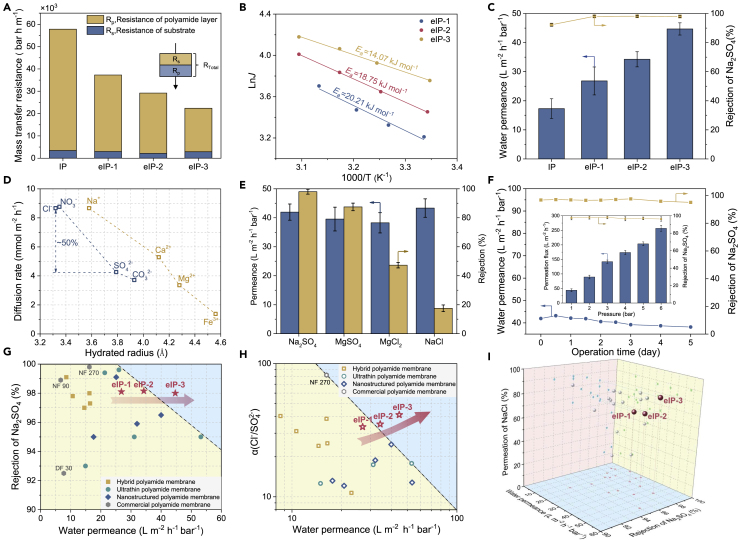
Permselectivity of polyamide membranes (A) Decoupled mass transfer resistance of polyamide membranes. (B) Activation energy for water transport of eIP polyamide membranes. (C) Water permeance and Na_2_SO_4_ rejection of polyamide membranes. (D) Size-dominant diffusion of hydrated ions across eIP polyamide membranes. Chloride salts and sodium salts were used to test the diffusion of cations and anions, respectively. (E) Permeance and salt rejection of the eIP-3 membrane. (F) Long-term crossflow desalination performance of the eIP-3 membrane. Inset: Desalination performance of the eIP-3 polyamide membrane under different driving pressure. (G−I) Comparison of nanofiltration performance of eIP membranes with state-of-the-art polyamide membranes.

Crossflow filtration is a technologically mature operation mode for industrial NF membranes. The cyclic flow allows for continuous filtration and alleviates the concentration polarization near the membrane surface but puts forward stringent requirements for the membrane's stability to withstand hydraulic shear (Morelos-Gomez et al., 2017). We established a lab-made crossflow filtration system to evaluate the membrane's separation performance (Figure S22). NF is widely applied to remove multivalent salts for water softening (Fang et al., 2014) and ionic separation (Cl^−^/SO_4_^2−^ mixture) (Zheng et al., 2017) bearing lower osmotic pressure. Therefore, the mass transfer resistance across the NF membrane accounts for the main energy consumption (Lim et al., 2016; Patel et al., 2020). We decoupled the membranes' mass transfer resistance in terms of the polyamide layer and substrate by the resistance-in-series model. As shown in Figure 4A, the PCS bears comparable resistance with the pristine PAN membrane due to the slightly increased water permeance of substrates (Figure S23). Besides, the polyamide layer predominates the overall transfer resistance of the composite membrane. Considering the similar hydrophilicity of resultant polyamide membranes (Figure S24), the relation between thickness and permeance/resistance can be established. With the reduction of membrane thickness from 51.0 ± 3.2 nm to 14.1 ± 1.3 nm, the water permeance of polyamide layer increased from 18.4 ± 2.6 L m^−2^ hr^−1^ bar^−1^ to 51.3 ± 1.8 L m^−2^ hr^−1^ bar^−1^ and the transfer resistance decreased from 55.9 ± 8.4 bar hr m^−1^ to 20.3 ± 0.7 bar hr m^−1^ correspondingly (Figure S25).

Insights into water transfer resistance can be obtained by Arrhenius activation energy (*E*_a_) from a thermodynamic perspective (Chen et al., 2018). As shown in Figure 4B, the reduced mass transfer resistance substantially decreases the activation energy of composite membranes down to 14.07 kJ mol^−1^, which is about 40%–50% lower than traditional polyamide and alginate polymer membrane (Cheng et al., 2019; Guan et al., 2018). Owing to the ultrathin and dense structure, the eIP-3 membrane displays (Figure 4C) superior water permanence of ~44.7 L m^−2^ hr^−1^ bar^−1^ along with a high Na_2_SO_4_ rejection of 98%. This performance is much better than the Fe^3+^-/NaCl-screened eIP-3 membranes and conventional IP membranes (Figure S26). Besides, the degenerated rejections of Fe^3+^-/NaCl-screened eIP-3 membranes reflect their less cross-linked structure. Additionally, although the eIP-1 and eIP-2 membranes feature a relatively looser structure and higher MWCO than the eIP-3 membrane, they exhibit a comparable rejection ratio (98.1%–98.2%). This high rejection is probably because the less cross-linked polyamide membrane has more -COO^−^ groups and thus is more negatively charged, which helps to reject SO_4_^2−^ ions by electrostatic repulsion (Figure S27) (Yuan et al., 2019).

To further explore the pemselectivity of eIP-3 membranes, we measured the diffusion rate of hydrated ions with varied chargeability and hydration radius (Figure S28). Figure 4D shows the decreased ion diffusion rates with the increased hydration radius in a typical size-dependent transport character, indicating that the steric hindrance dominates the ionic selectivity of the membrane. According to the fitting curve, we also found that the anion diffusion rate is much slower than the cation diffusion rate when bearing a similar hydration radius. This divergent diffusion behavior may be ascribed to the electrostatic repulsion between negatively charged polyamide and anions as mentioned above (Figure S27A). Based on the selective ionic diffusion, the eIP-3 membrane offers potential for ionic separation application. Then, we found the desalination performance of eIP-3 membranes toward different kinds of salts with rejection in the order of Na_2_SO_4_>MgSO_4_>MgCl_2_>NaCl, which implies a synergistic influence of steric hindrance and electrostatic repulsion as a typical polyamide membrane (Wang et al., 2018). Benefited from the robust substrate based on the covalent-like metal-organophosphate bond and highly cross-linked polyamide network, the eIP-3 membrane maintains desirable desalination efficiency during 5-day crossflow filtration with a hydraulic shear speed of 40 L hr^−1^ and withstands varied driving pressure and a wide range of salt concentration (Figures 4F and S29). Additionally, by applying eIP-3 in organic solvent nanofiltration, the membrane can maintain molecular selectivity in ethanol and remove organic dyes from 452 Da to 960 Da, demonstrating potential in molecular separation (Figures S30 and S31). This structural durability of the eIP-3 membrane makes it qualified for practical application.

The current eIP strategy generates ultrathin and highly cross-linked polyamide membranes, offering an effective way to breaking the trade-off effect between water permanence and Na_2_SO_4_ rejection among traditional polyamide (Figure 4G). As shown in Figure 4H, the eIP-3 membrane also exhibits outstanding permselectivity for the challenging sub-nanoscale separation of Cl^−^ and SO_4_^2−^ arising from the ~50% slower diffusion rate of SO_4_^2−^ than Cl^−^ (Cl^−^/SO_4_^2−^ selectivity of 41.2, Figure 4D). This high selectivity contributes to both high rejection of Na_2_SO_4_ (98.0%) and permeation of NaCl (82.5%) along with an ultrafast water permeation (Figure 4I). As the pre-treatment of reverse osmosis, such high NaCl permeation can significantly decrease the osmotic pressure and reduce the energy intensity of the nanofiltration process. To further demonstrate the technological advancement of eIP, we compared the eIP membranes with the commercial benchmark polyamide membranes, including NF270 (DOW), NF90 (DOW), and DF30 (OriginWater) in our lab-made crossflow system. As shown in Figures 4G−4I, the eIP membranes display superior performance in water desalination and ionic separation (Figures 4G−4I and Table S4). Given that the practical salty water is usually a mixture of salts, we conducted ionic separation performance with mixed salt solution containing Na_2_SO_4_ and NaCl (total concentration of 2000 ppm and 4000 ppm). Figure S32 shows that the eIP-3 membrane could remove ~95% SO_4_^2−^ from the mixed salt solution. The considerably increased ionic strength probably accounts for the slightly decreased SO_4_^2−^ from 98% to 95%. Intriguingly, the NaCl was 8%–10% enriched in the permeates after filtration. This abnormal NaCl concentration effect is because of the co-ion competition effect between Cl^−^ and SO_4_^2−^ facilitating the transport of Cl^−^ (Luo and Wan, 2013). From the practical perspective of resource reuse, high SO_4_^2−^ rejection along with high Cl^−^ permeation counts more than the single metric of Cl^−^/SO_4_^2−^ selectivity (Hao et al., 2020; Sarkar et al., 2020; Zhu et al., 2018). Conclusively, the unprecedented ionic separation performance endows the eIP-3 membrane with great promise for brine refinement and salt reclamation applications (Sarkar et al., 2020).

## Discussion

In summary, we proposed an eIP approach for the highly controllable fabrication of ultra-permselective polyamide membranes. The phytate molecule bearing superior charge density is coordinatively anchored onto a porous UF membrane as a supercharged substrate, conferring high-density long-range electrostatic interaction to attract amine monomers. By varying the phosphate content from flexible coordination, the surface charge density of the PCS spanning from −4.84 mC m^−2^ to −6.02 mC m^−2^ harvests 7-nm-range electrostatic interaction with PIP to increase amine concentration and retard amine diffusion into organic phase to polymerize with TMC. The enriched PIP on the supercharged PCS triggers the self-sealing and increases the cross-linking degree of as-prepared polyamide membrane to 70%–78%, superior to conventional IP-based polyamide membranes (40%–60%). Besides, the retarded PIP diffusion leads to a lower transport rate across the aqueous-organic interface. Therefore, the eIP generates highly cross-linked polyamide membranes with tunable thickness down to 14 nm within merely 15 s, 3–7 times faster than the conventional IP process. By further screening electrostatic attraction, the enrichment and retarded diffusion effects were considerably weakened accompanying by thicker and less cross-linked membrane structure, elucidating that both high charge density and long interaction range contribute to the eIP process. The optimized eIP membrane displays nanofiltration efficiency with water permeance of 44.7 L m^−2^ hr^−1^ bar^−1^ and a high Cl^−^/SO_4_^2−^ selectivity of 41.2 (Na_2_SO_4_ rejection of 98% and NaCl permeation of 82.5%), outperforming the benchmark commercial membranes and reported state-of-the-art polyamide membranes. In principle, eIP technology is applicable to the majority of conventional IP processes and can be extended to fabricate a broad range of IP-based ultrathin membrane materials, such as emergent MOFs and COFs.

### Limitations of the study

Considering the supercharged metal-organophosphate layer bearing limited porosity, which has been demonstrated to be effective in monomer storage (Yuan et al., 2019), further advances in eIP could be achieved by engineering supercharged porous materials like recently reported ionic covalent organic frameworks (iCOFs). These iCOFs with pre-designable and diversiform charged groups could offer either negative (Cao et al., 2020) or positive (He et al., 2020) chargeability to electrostatically regulate both conventional positively charged amine monomers toward polyamide membranes and emergent negatively charged hydroxy monomers toward polyester membranes (Shen et al., 2021).

### Resource availability

#### Lead contact

Further information and requests for resources and reagents should be directed to and will be fulfilled by the lead contact, Zhongyi Jiang (zhyjiang@tju.edu.cn).

#### Materials availability

This study did not generate new unique reagents.

#### Data and code availability

This study did not generate/analyze data sets/code. All data are described in the main text and all analysis methods in the supplemental information.

## Methods

All methods can be found in the accompanying transparent methods supplemental file.

## Supplemental information

Document S1. Transparent methods, Figures S1–S32, and Tables S1–S4

## Footnotes

Supplemental information can be found online at https://doi.org/10.1016/j.isci.2021.102369.
