# Supplementary material for: Electrostatic-modulated interfacial polymerization toward ultra-permselective nanofiltration membranes
Source: iScience. 2021 Mar 26;24(4):102369. doi: 10.1016/j.isci.2021.102369 (PMC8059057; doi:10.1016/j.isci.2021.102369)
Supplement: Document S1. Transparent methods, Figures S1–S32, and Tables S1–S4 [file mmc1.pdf]

iScience, Volume *24*

Supplemental information

Electrostatic-modulated interfacial polymerization toward ultra-permselective nanofiltration membranes

Xinda You, Ke Xiao, Hong Wu, Yafei Li, Runlai Li, Jinqiu Yuan, Runnan Zhang, Zhiming Zhang, Xu Liang, Jianliang Shen, and Zhongyi Jiang

1. Supplemental Figures

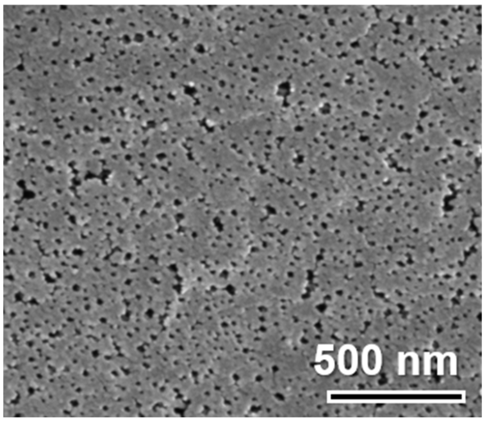

Figure S1. SEM image of polyacrylonitrile (PAN) membrane. The PAN susbtrate was air-dried before SEM characterization. Related to Figure 2.

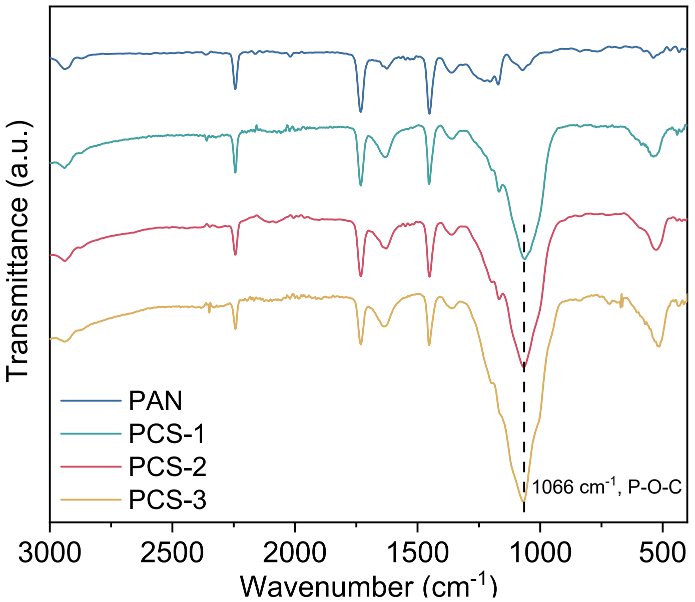

Figure S2. FTIR spectra of PAN substrate and PCSs. The Attenuated Total Reflectance mode was utilized for FTIR. Related to Figure 2.

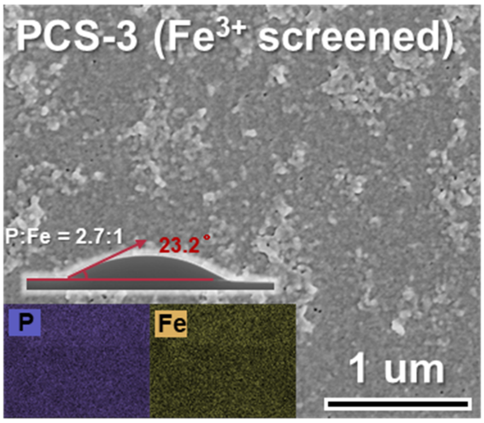

Figure S3. SEM image of PCS-3 screened by Fe^{3+}. Insets: elemental distribution and water contact angle. Related to Figure 2.

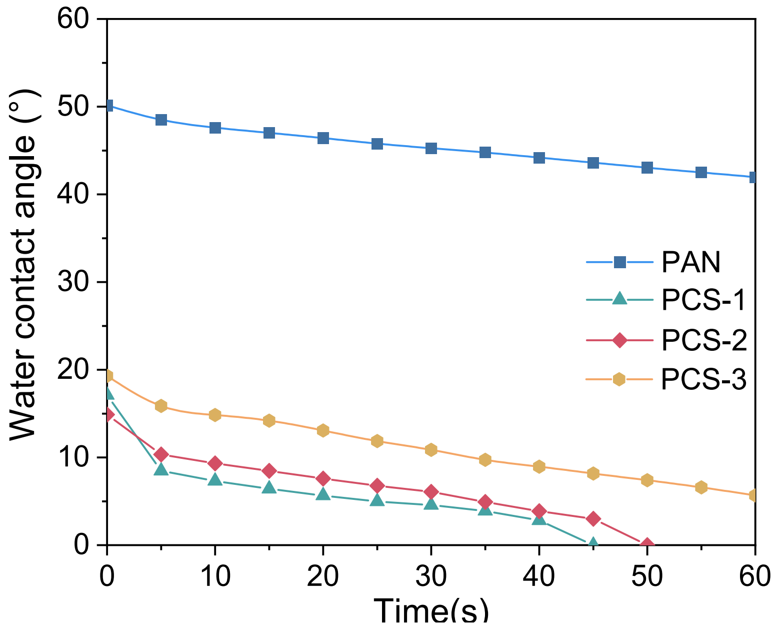

Figure S4. Dynamic water contact angles of PAN membrane and PCSs. The stopping time was fixed at 60 s. Related to Figure 2.

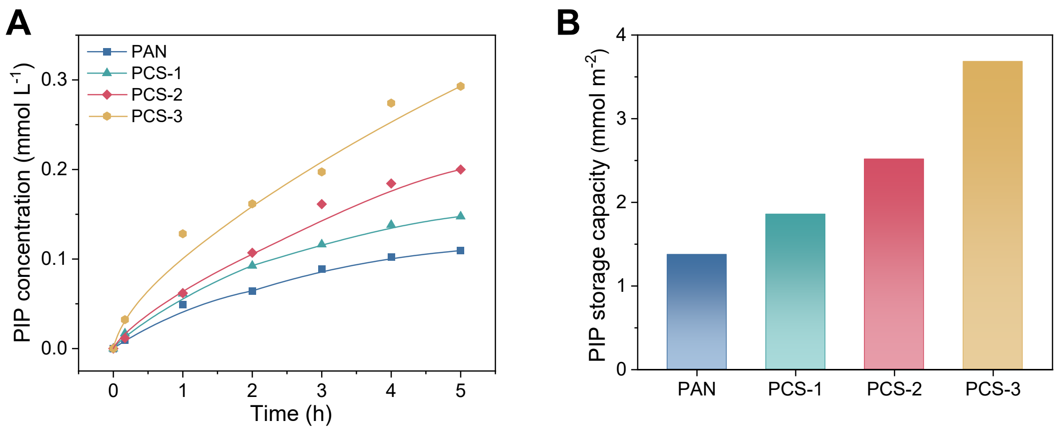

Figure S5. PIP diffusion and sotrage properties and subtrates. (A) Detected PIP diffusion from substrate to organic phase. (B) PIP storage capacity of substrates. Related to Figure 2.

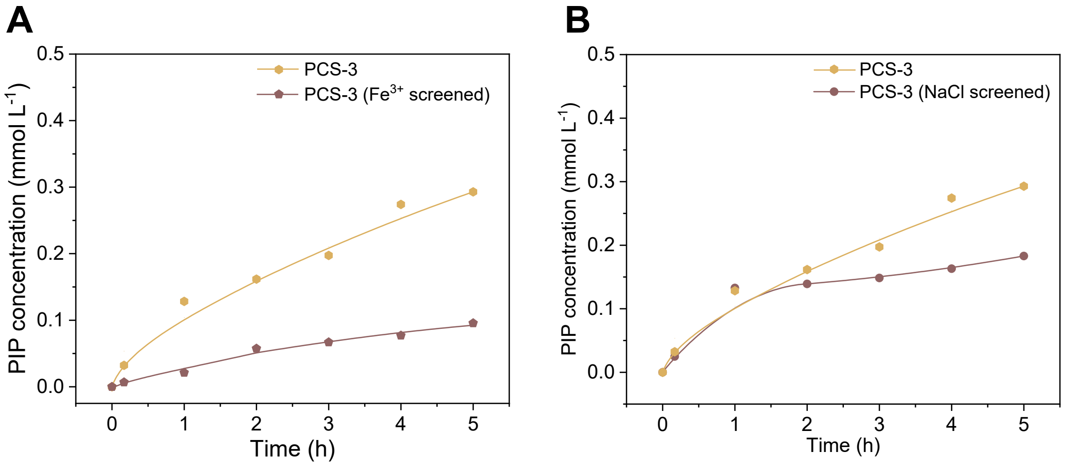

Figure S6. PIP diffusion behavior of PCS-3 substrates. Detected PIP diffusion from the substrate to the organic phase of (A) Fe^{3+}-screened and (B) NaCl-screened PCS-3. Related to Figure 2.

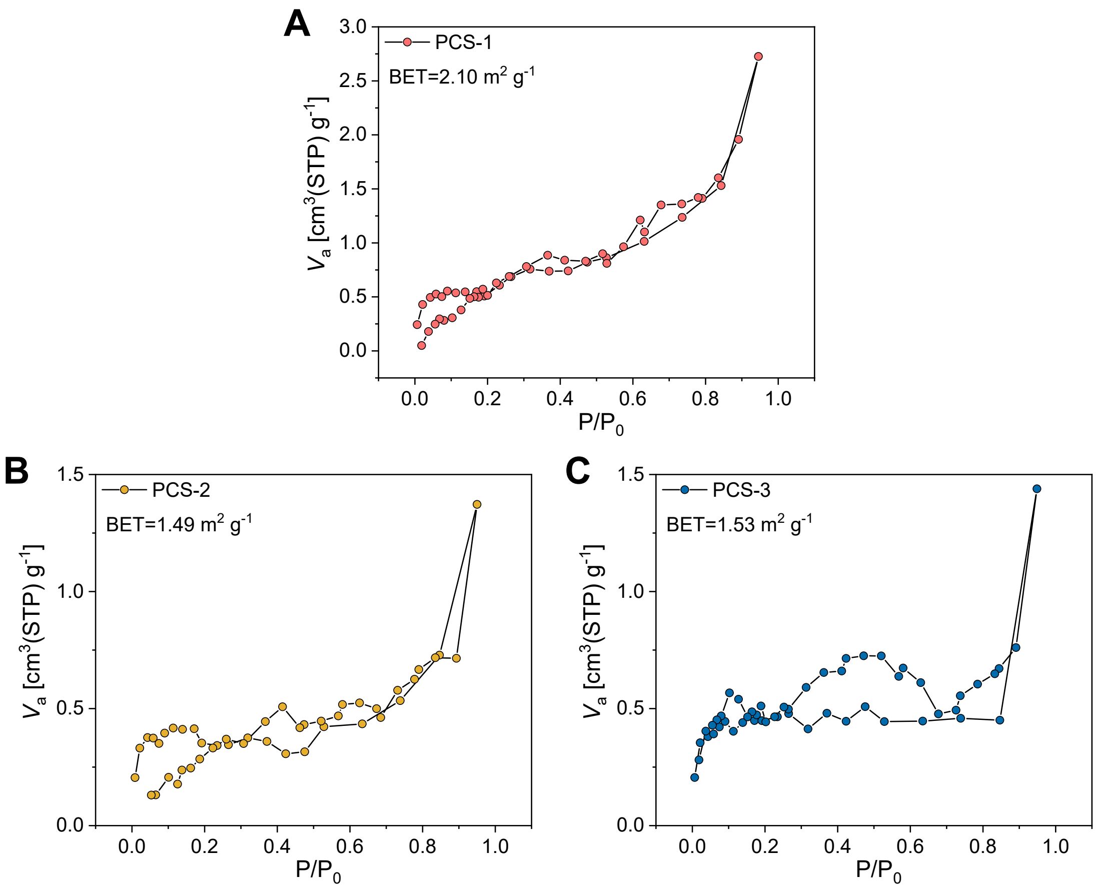

Figure S7. Porosity of PCSs measured by BET method. BET surface area of (A) PCS-1, (B) PCS-2 and (C) PCS-3. Related to Figure 2.

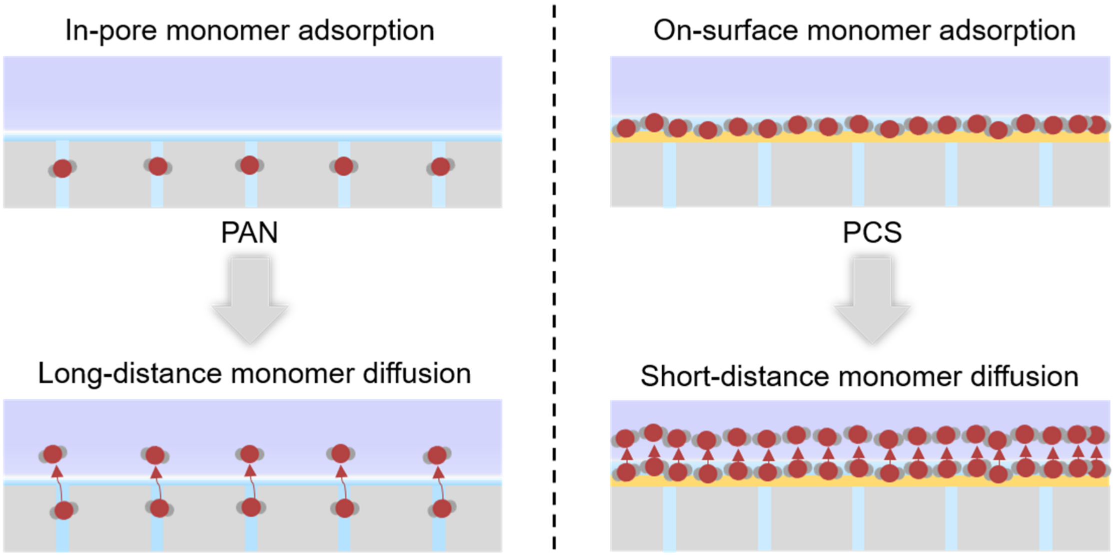

Figure S8. The PIP monomer adsorption and diffusion behavior of PAN and PCS. The PAN features in-pore monomer storage while PCS features on-surface monomer storage. Related to Figure 2.

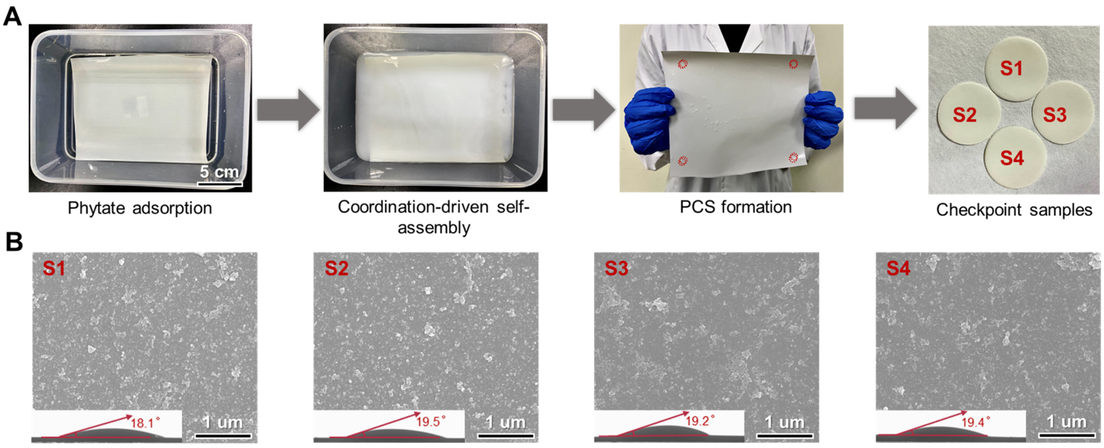

Figure S9. Large-scale fabrication and detection of PCS-3. (A) Scale-up fabrication of PCS-3. (B) SEM images of different checkpoint samples of PCS-3. PAN membrane size: 15 cm×21 cm=315 cm^2=0.0315 m^2. Assembly solution: (6.75 mL PA solution+382.5 mL DI water)+(1890 mg $FeCl_3$ $6H_2O$+67.5 mL). Related to Figure 2.

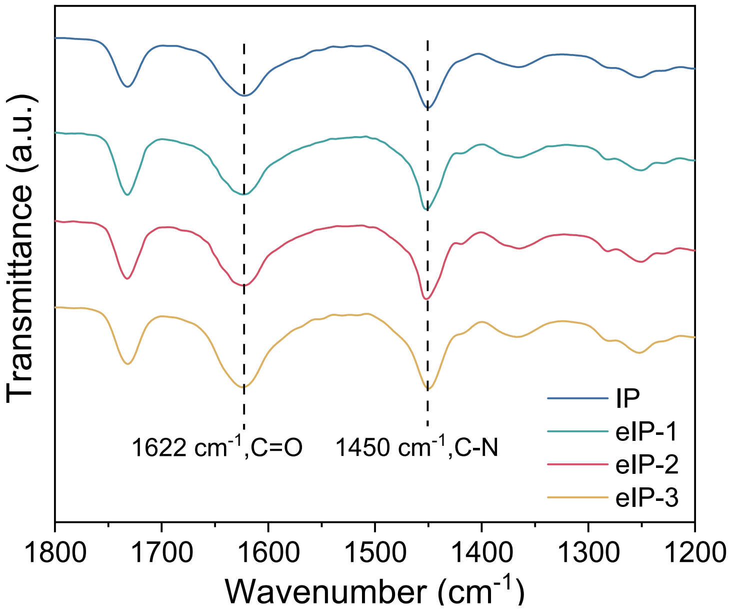

Figure S10. FTIR spectra of polyamide membranes prepared by conventional IP and eIP. The Attenuated Total Reflectance mode was utilized for FTIR. Related to Figure 3.

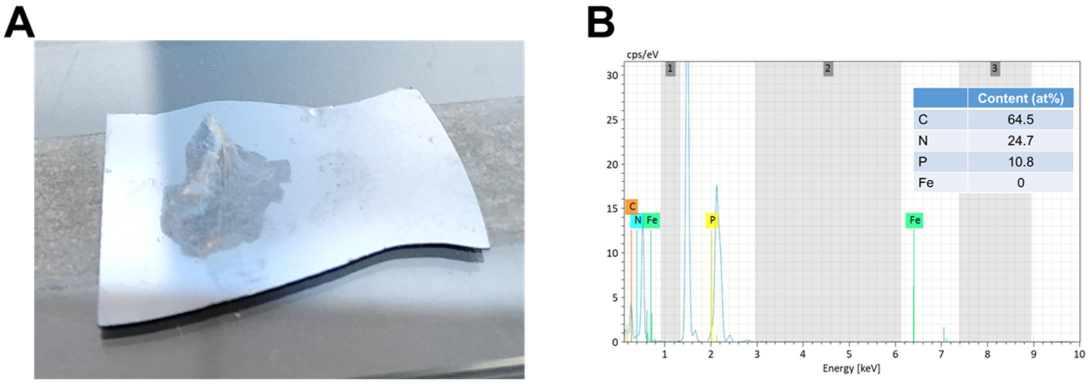

Figure S11. Macrostructure and EDS results of substrate-free polyamide membrane. (A) Substrate-free polyamide membrane on silica plate. (B) Elemental composition of substrate-free eIP polyamide membrane by EDS. As shown in Supplementary Figure 11 (B), the Fe elemental content of the substrate-free polyamide membrane is 0%, verifying the phytate-Fe^{3+} network is disassembled by DMF and ethanol. The detected P element is probably caused by the adsorption of phytate. This molecule-level adsorption would not influence the thickness of the polyamide membrane. Related to Figure 3.

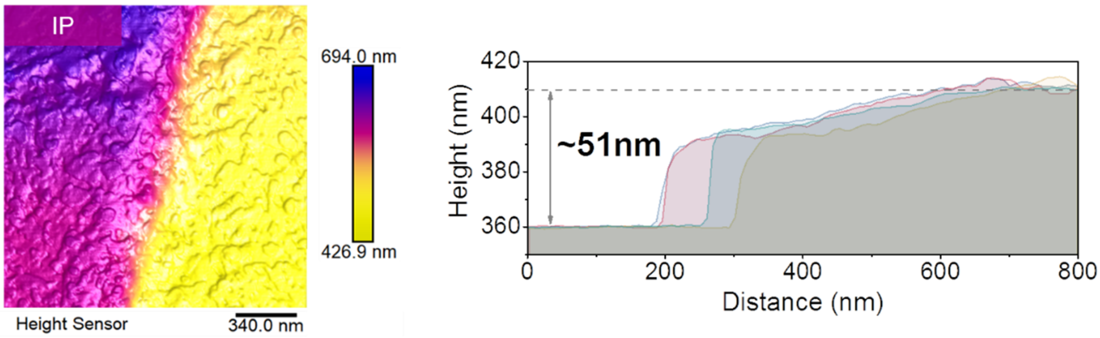

Figure S12. AFM image and related height profile of substrate-free polyamide membrane prepared by the conventional IP. Related to Figure 3.

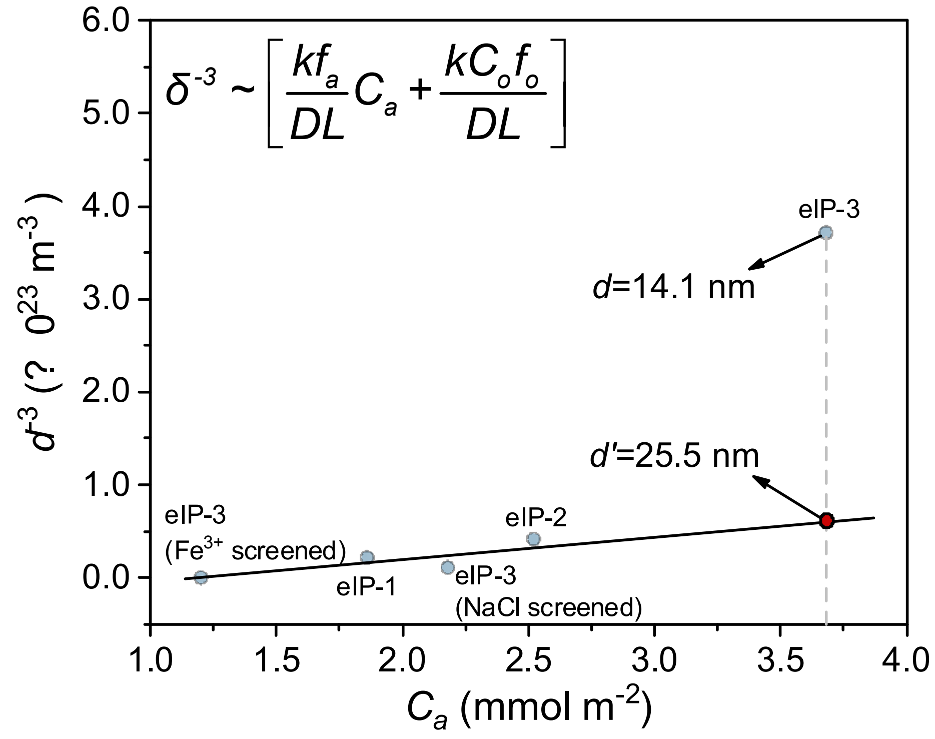

Figure S13. **Correlation between membrane thickness and PIP concentration by transformed Freger's model.** Considering the C_a is directly proportional to PIP storage capacity, we used the latter to represent the former for quantitative analysis. The thickness of the screened eIP membrane was obtained from AFM height profiles in Supplementary Figure 16. Theoretically, the δ^{-3} is linearly dependent on C_a. However, the thickness of eIP-3 membrane is substantially lower than the predicted value, indicating that the electrostatic-retarded monomer diffusion of PCS-3 also contributes to the reduced membrane thickness. Related to Figure 3.

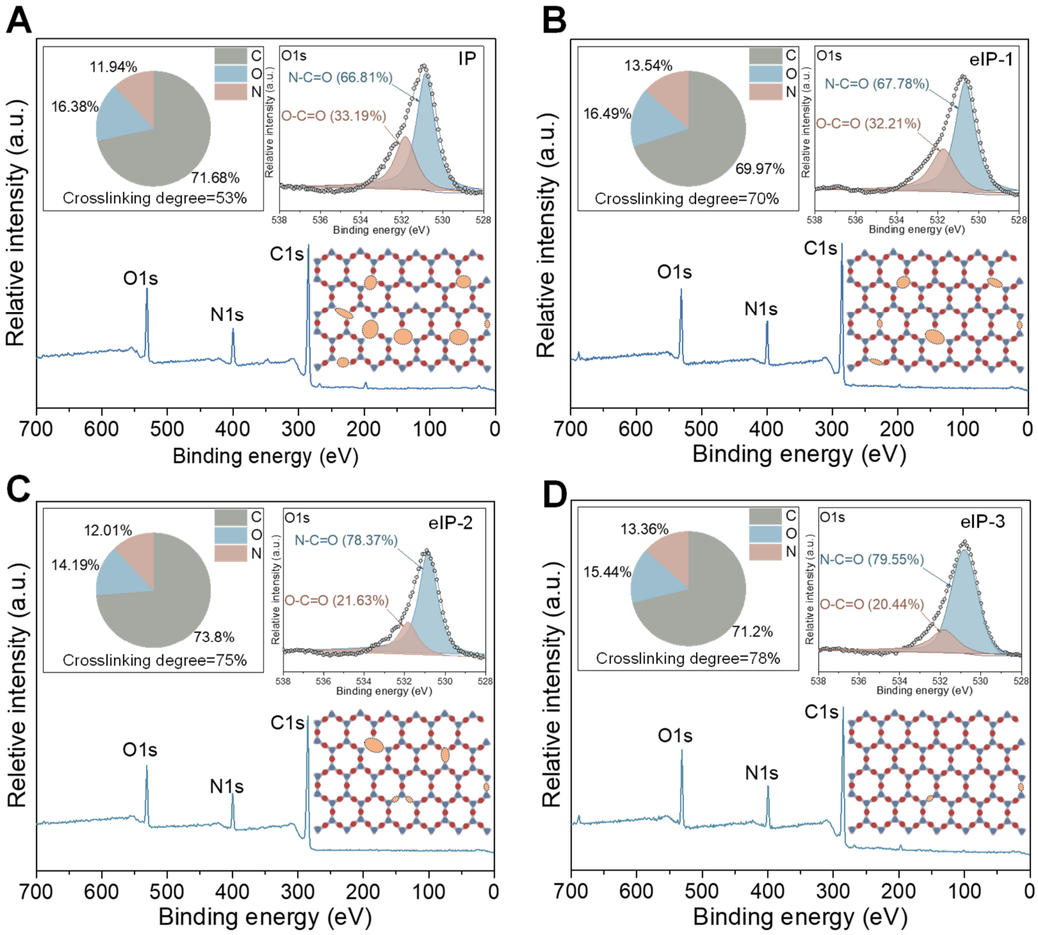

Figure S14. XPS spectra of polyamide membranes. Insets: Elemental composition (top left), high-resolution O1s-core spectrum (top right) and crosslinking structure (bottom right) of polyamide membranes. The crosslinking degree (D, %) of the polyamide membrane was calculated as follows:

$$\frac{O}{N}=\frac{3m+4n}{3m+2n} \tag{1}$$

$$D=\frac{m}{m+n}\times 100\% \tag{2}$$

where m and n are the crosslinked and linear proportion of the polyamide, respectively. Related to Figure 3.

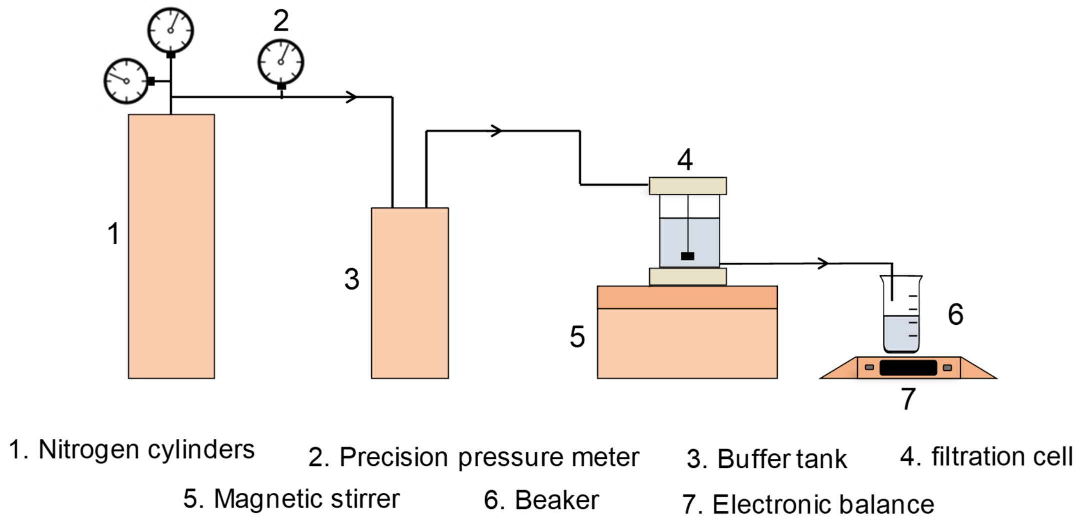

Figure S15. Schematic diagram of the dead-end filtration apparatus for testing MWCO by organic solvent nanofiltration. Related to Figure 4.

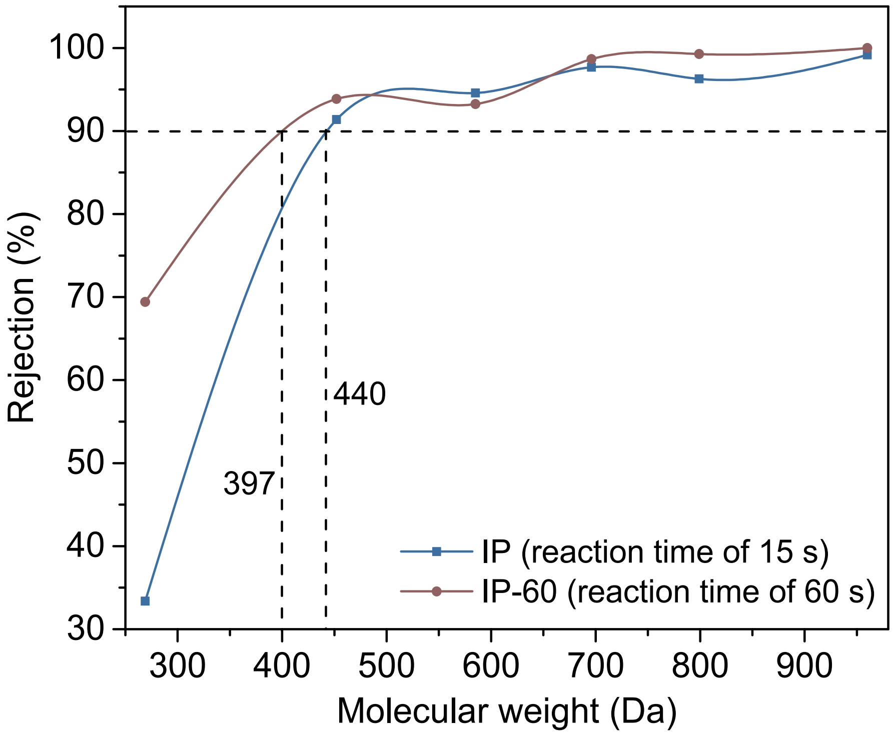

Figure S16. Molecular weight cut-off (MWCO) of polyamide membranes prepared by conventional IP with different reaction times. The MWCO was measured by organic solvent nanofiltration with organic dyes as solutes and ethanol as solvent. Related to Figure 4.

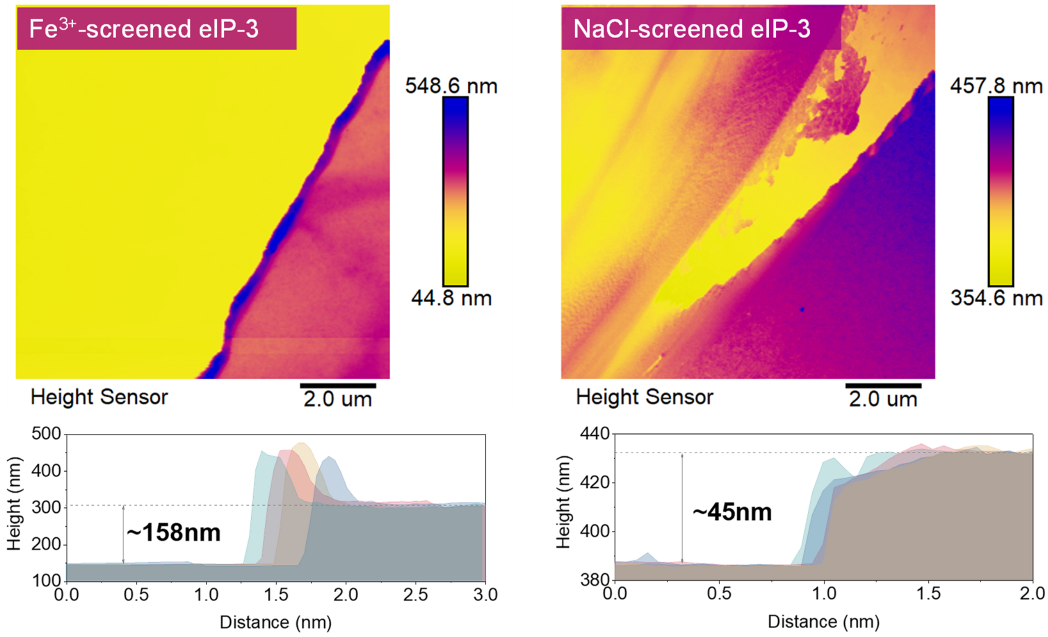

Figure S17. AFM images and related height profiles of substrate-free eIP-3 polyamide membranes fabricated on Fe^{3+}- screened and NaCl-screened PCS-3. Related to Figure 4.

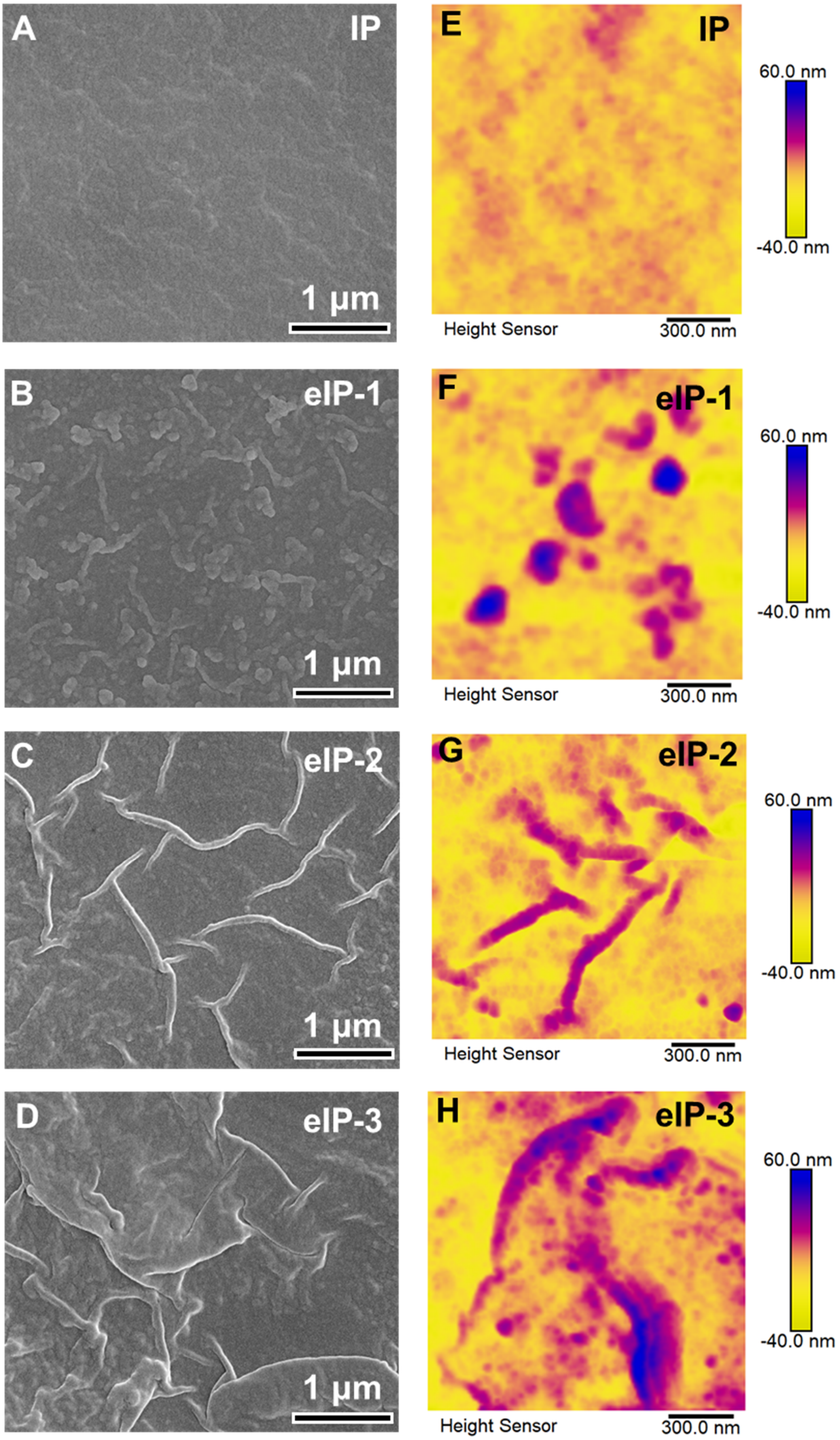

Figure S18. Morphology of polyamide membranes. (A−D) SEM images and (E−H) AFM images of polyamide membranes prepared by conventional IP and eIP. Related to Figure 4.

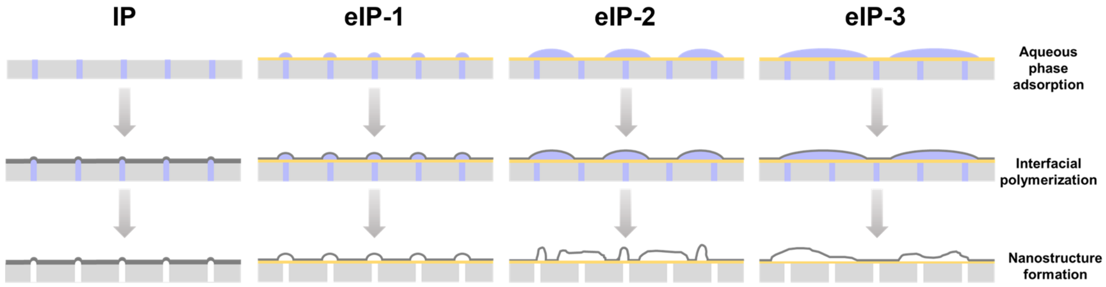

Figure S19. Schematic diagram of the formation of surface nanostructures by water template. Related to Figure 4.

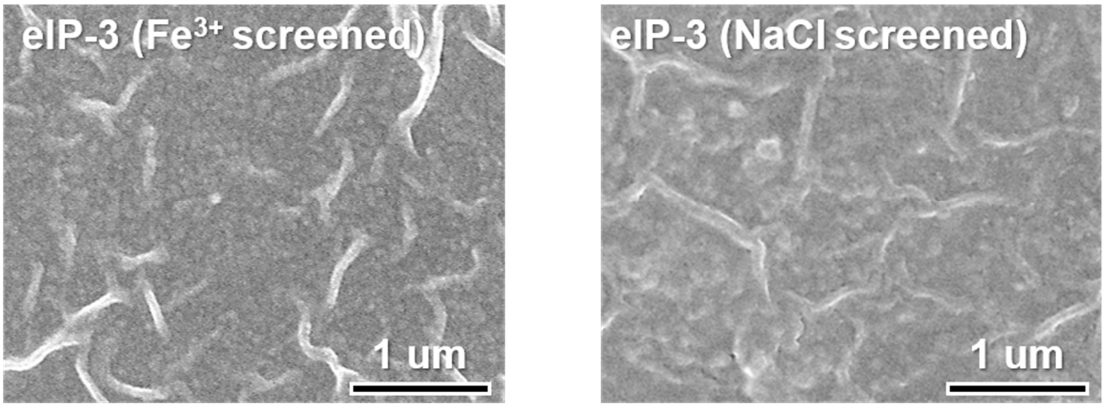

Figure S20. SEM images of eIP-3 polyamide membranes fabricated on Fe^{3+}- screened and NaCl-screened PCS-3. Related to Figure 4.

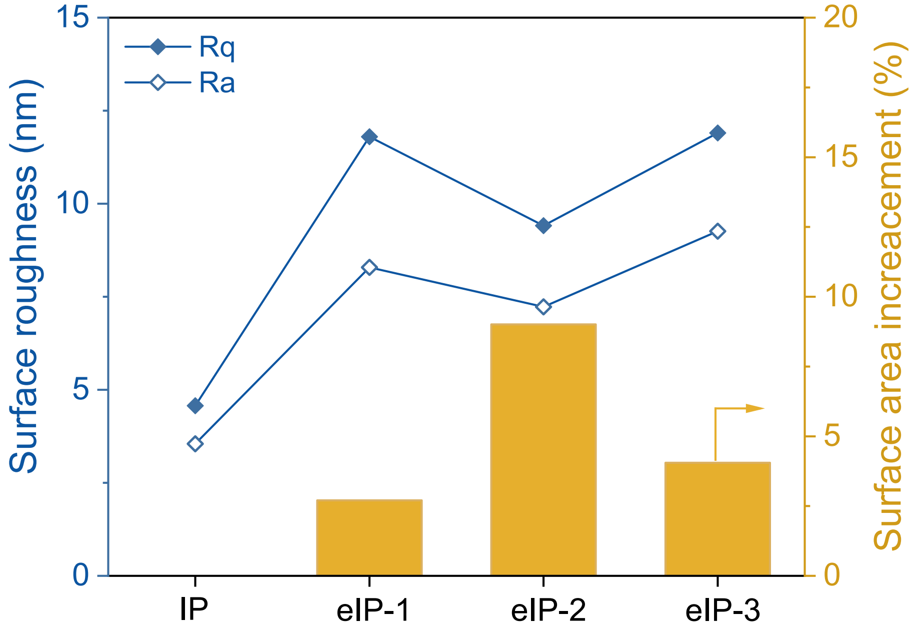

Figure S21. Surface roughness and surface area increasement of polyamide membranes prepared by conventional IP and eIP. Related to Figure 4.

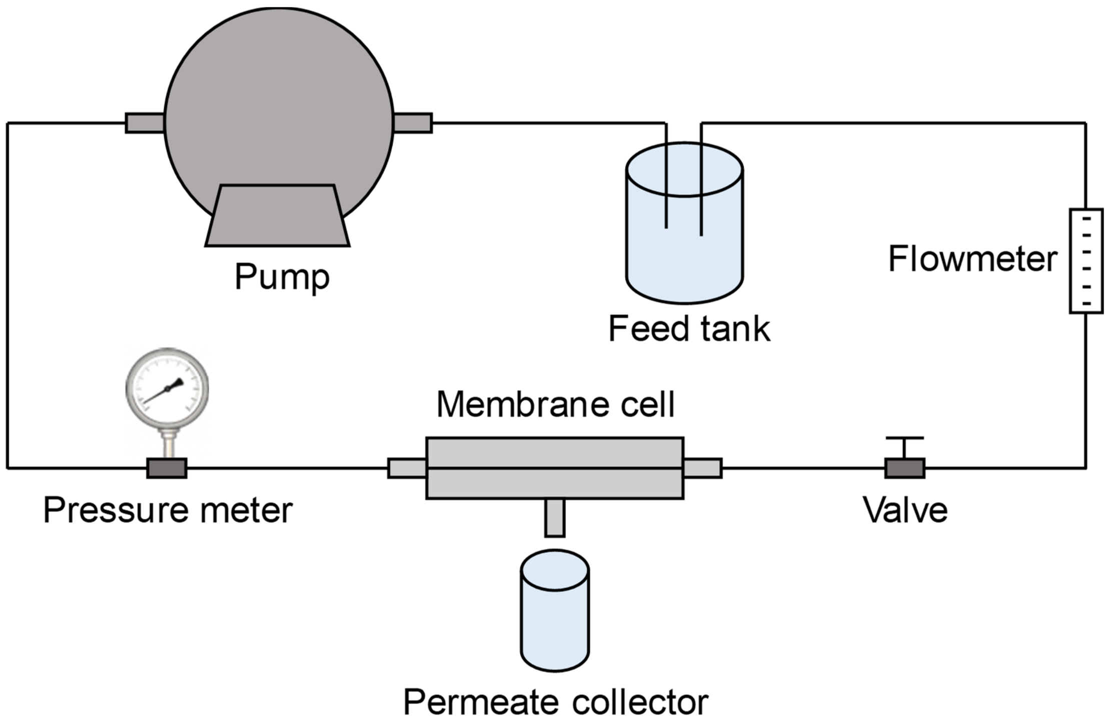

Figure S22. Schematic diagram of the crossflow filtration apparatus for desalination. Related to Figure 4.

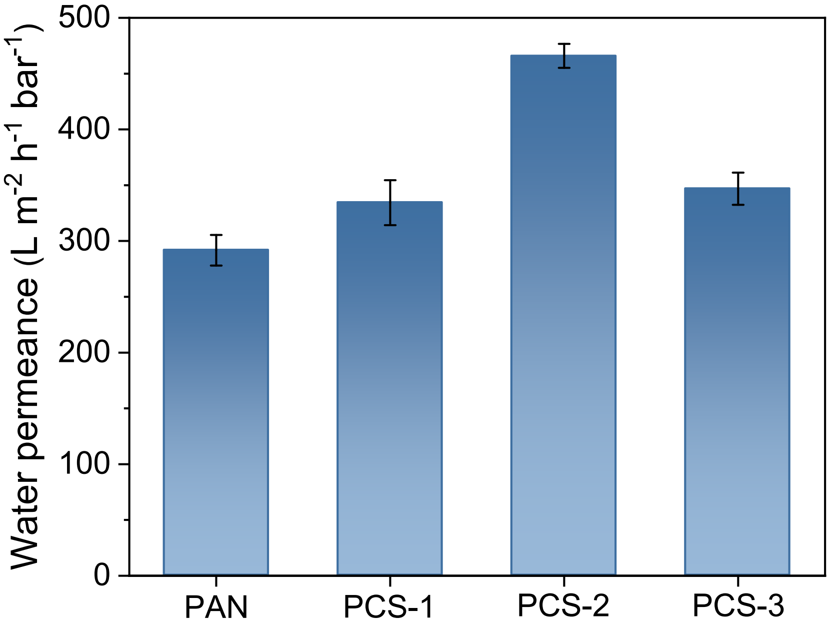

Figure S23. Pure water permeance of PAN membrane and PCSs. The error bars indicate s.d. Related to Figure 4.

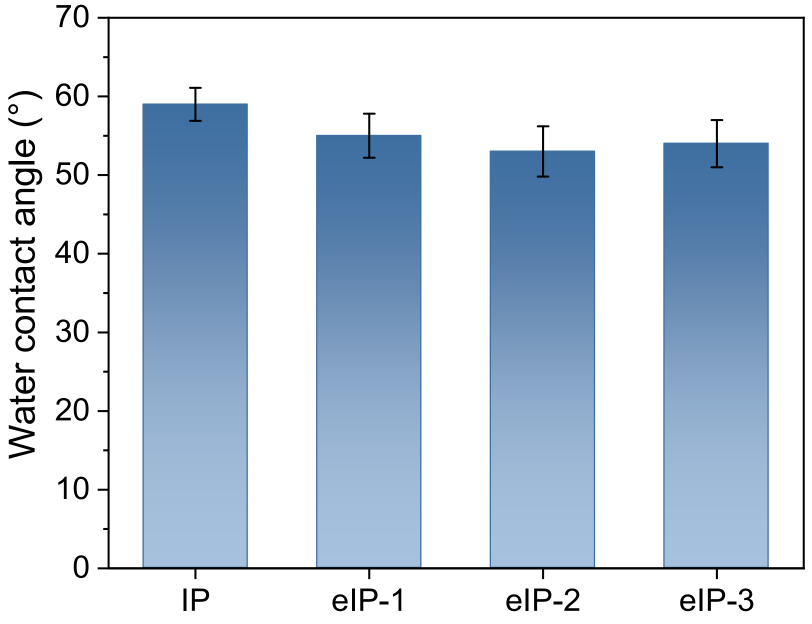

Figure S24. Water contact angles of polyamide membranes prepared by conventional IP and eIP. The error bars indicate s.d. Related to Figure 4.

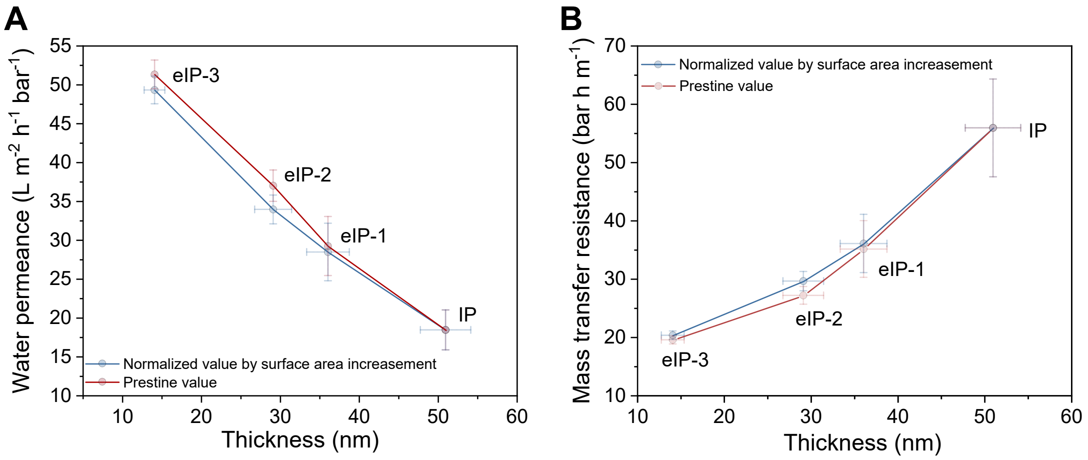

Figure S25. Corelation between membrane thickness and water transport. (A) Thickness-dependent water permeance of the polyamide layer. (B) Thickness-dependent transfer resistance of polyamide layer. The error bars indicate s.d. The blue line is the normalized value, excluding the influence of increased surface area from nanostructure. The impact of the substrate was also excluded by the resistance-in-series model. Related to Figure 4.

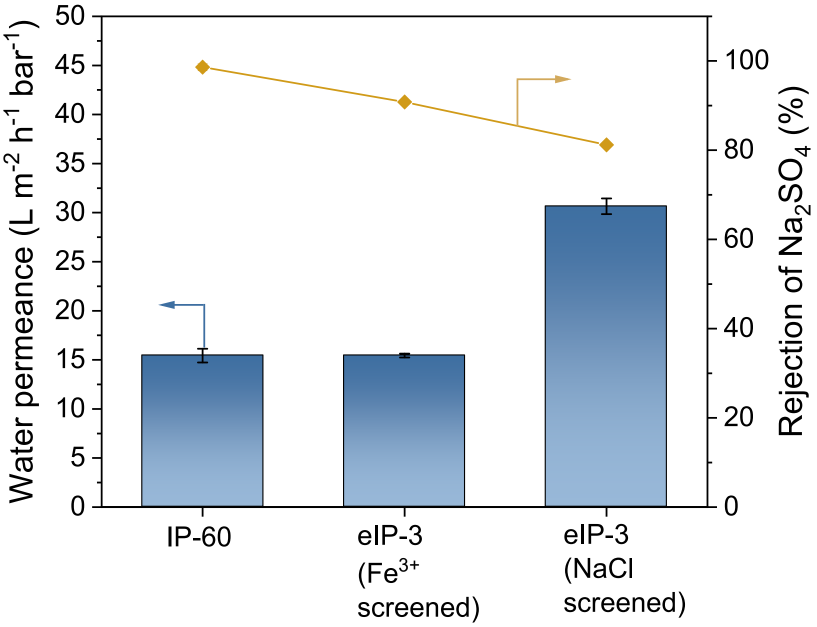

Figure S26. Water permeance and salt rejection of eIP-3 fabricated on Fe^{3+}-screened and NaCl-screened PCS-3. The error bars indicate s.d. Related to Figure 4.

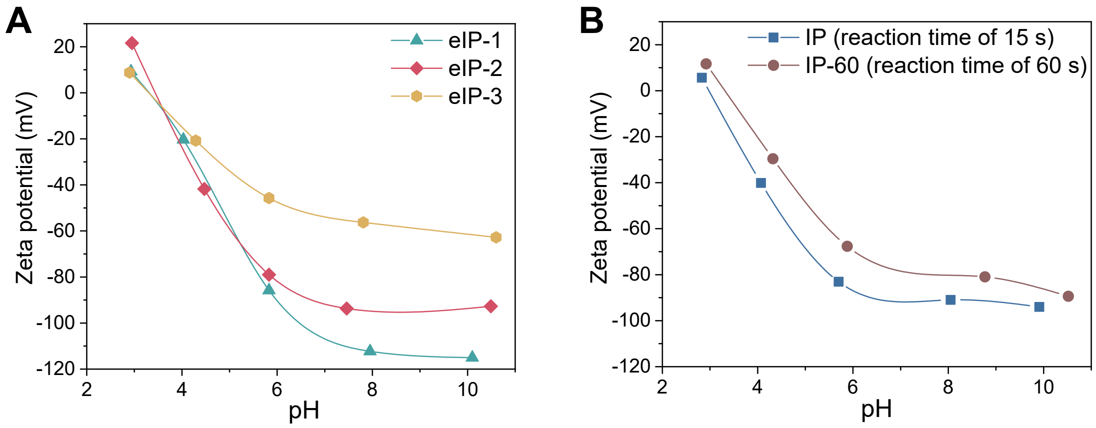

Figure S27. Zeta potentiao of polyamide membranes. (A) pH-dependent surface zeta potential of polyamide membranes prepared by eIP. (B) pH-dependent surface zeta potential of polyamide membranes prepared by conventional IP with different reaction times. Related to Figure 4.

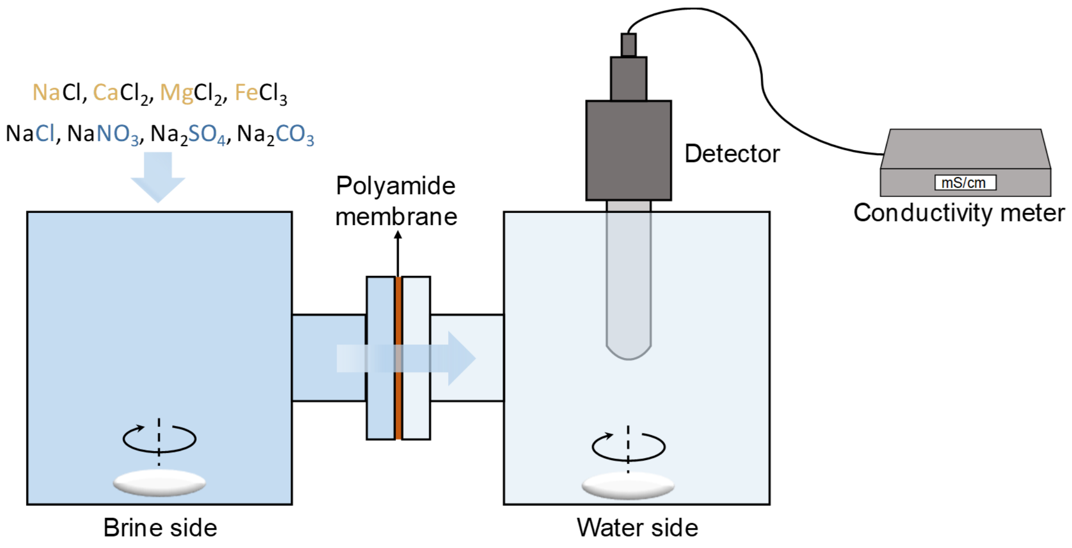

Figure S28. Schematic diagram of H-shape diffusion cell for ionic diffusion measurement. Related to Figure 4.

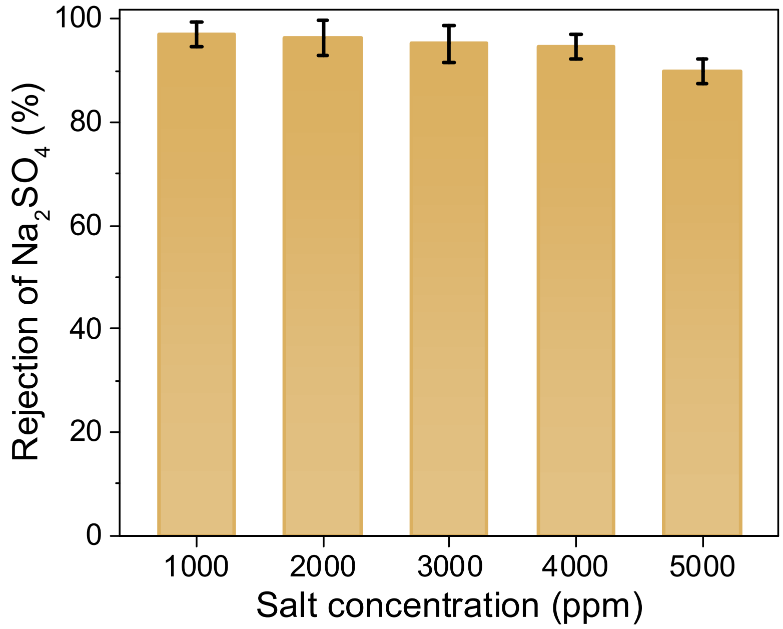

Figure S29. Na_2SO_4 rejections of eIP-3 polyamide membranes with different salt concentrations. The error bars indicate s.d. Related to Figure 4.

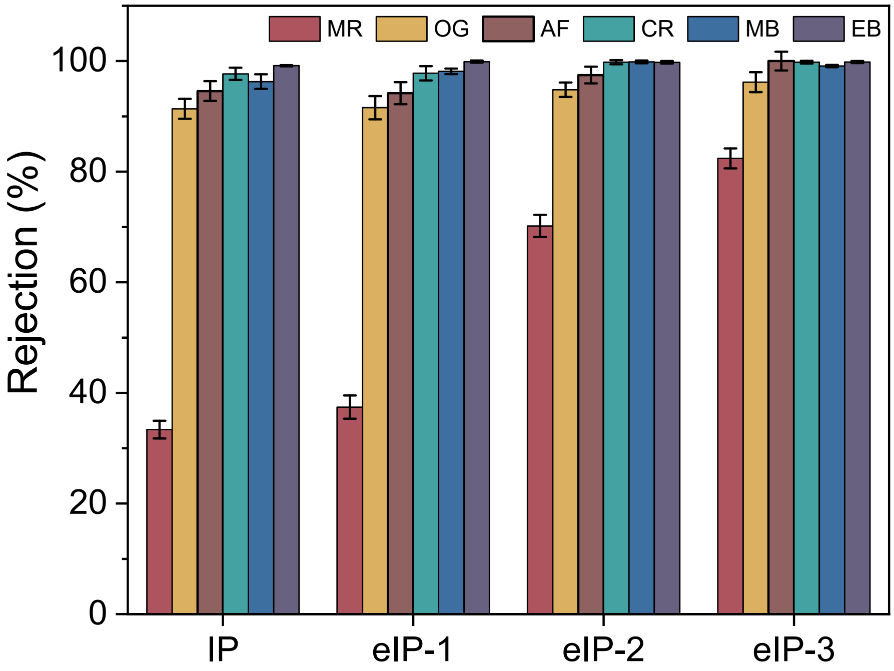

Figure S30. Organic solvent nanofiltration performance of polyamide membrane prepared by conventional IP and eIP. (Solvent: ethanol, dye concentration: 100 ppm). The error bars indicate s.d. Related to Figure 4.

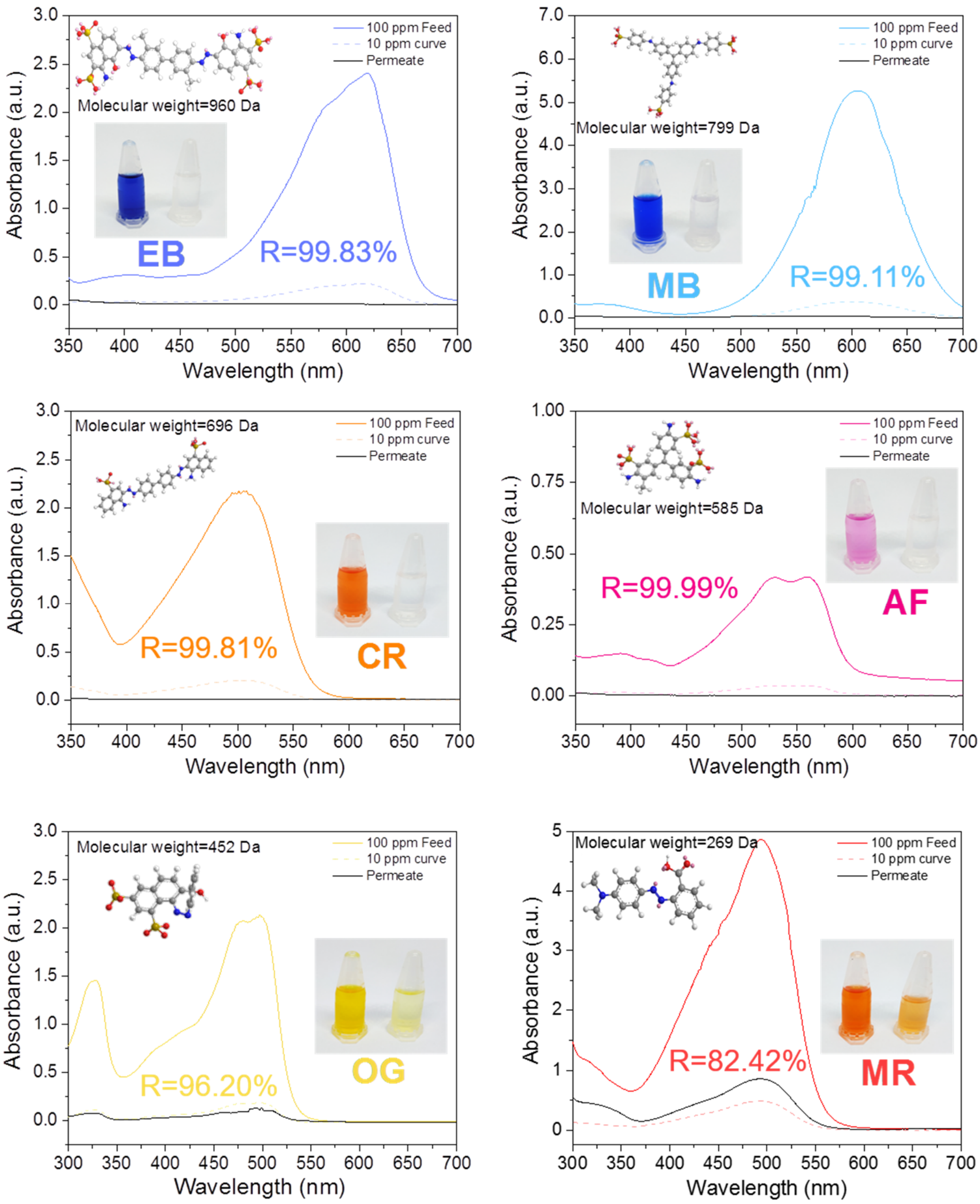

Figure S31. Ultraviolet-visible absorption spectra of dyes in feed and filtrate of eIP-3 polyamide membrane. Insets: Stereochemical structure of organic dyes and digital photo images of feed and permeate. (Solvent: ethanol, dye concentration: 100 ppm). Related to Figure 4.

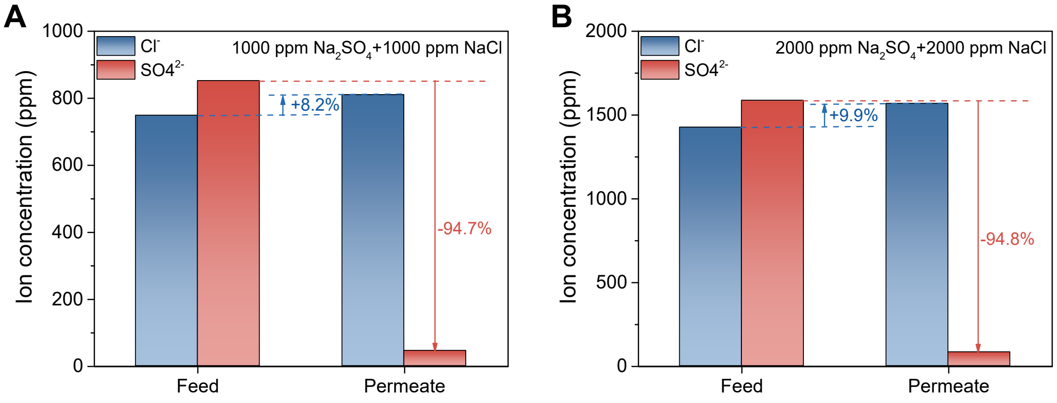

Figure S32. Ionic separation performance of eIP-3 polyamide membrane with different salt concentrations and composition. Related to Figure 4.

2. Supplemental Tables

Table S1. Summary of relative monomer diffusion rate (D_r) of different substrates. Related to Figure 2.

Substrate name	D_r (h^{-1})
PAN	0.509
PCS-1	0.741
PCS-2	0.730
PCS-3	0.655
PCS-3 (Fe^{3+} screened)	0.792
PCS-3 (NaCl screened)	0.854

Table S2. Fabrication parameters for membranes. Related to Figure 3.

Membrane name	Phytate concentration (g L^{-1})	PIP concentration (g L^{-1})	TMC concentration (g L^{-1})	Reaction time (s)
IP	0	0.875	0.670	15
IP-60	0	0.875	0.670	60
eIP-1	4.56	0.875	0.670	15
eIP-2	9.12	0.875	0.670	15
eIP-3	18.24	0.875	0.670	15

Table S3. Summary of reaction time and crosslinking degree of polyamide membranes in literature. Related to Figure 3.

Membrane name	Reaction time (s)	Crosslinking degree (%)	Ref.
IP	15	53	This work
eIP-1	15	70	
eIP-2	15	75	
eIP-3	15	78	
TFN-mZIF	60	50	(Zhu et al., 2017)
Ts-II	60	53	(Tan et al., 2018)
PA-ATP/PES	120	47	(Wu et al., 2017)
PA/PDA-COF/PAN	120	55	(Wu et al., 2019)
PA/CLS	120	59	(Yuan et al., 2019)
PEG-POSS-PA	120	61	(You et al., 2017)

Table S4. Summary of water desalination and ionic separation performance of polyamide membranes (Crossflow filtration mode). Related to Figure 4.

Membrane name	Water permeance ($L\ m^{-2}\ h^{-1}\ bar^{-1}$)	Na_2SO_4 rejection (%)	NaCl recovery (%)	Cl^-/SO_4^{2-} selectivity	Ref.
eIP-3	44.7	98.0	82.5	41.2	
Commercial polyamide membranes					
NF 270 (DOW®)	16.2	99.8	16.4	82	Test in this work
NF 90 (DOW®)	6.7	98.9	12.8	79.3	
DF 30 (OriginWater®)	7.7	92.5	N/A	N/A	
Hybrid polyamide membranes					
PIP/Sericin-TMC NF2	16.4	97.3	68	25.2	(Pan et al., 2017)
CNC-TFC	16.2	98	77	38.5	(Bai et al., 2019)
TFC2.0-5	14.5	97	72.3	24.1	(Zhu et al., 2018b)
ZNGTFNM2	10.6	97.8	68.4	31.1	(Ji et al., 2018)
PA-PPTA/PSf	8.52	99.1	36.4	44.4	(Shi et al., 2017)
Ultrathin polyamide membranes					
PA/SWCNT	40.0	96.5	86.6	24.7	(Gao et al., 2019)
PA/PD/SWCNTs	32.0	95.9	77	18.8	(Zhu et al., 2016)
NCMs	25.1	99.1	72.5	80.6	(Zhu et al., 2018a)
TFC NFMs	17.6	95	66	13.2	(Wu et al., 2016)
Nanostructured polyamide membranes					
PA/PD/ZIF-8/SWCNTs	53.2	95.0	89	17.8	(Wang et al., 2018)

PA/COFs	31.1	95	87	17.4	(Zhang et al., 2019)
Ts-II	26.0	99.6	50.4	126	(Tan et al., 2018)
TFC-R	21.3	99.4	N/A	N/A	(Jiang et al., 2019)
TFN-mZIF	14.9	93.0	88.5	12.6	(Zhu et al., 2017)

3. Transparent Methods

Materials

Polyacrylonitrile (PAN) ultrafiltration membrane (MWCO=100 kDa) was purchased from Lanjing Membrane Engineering Co. (Shandong, China). Phytate (50wt% in water) was purchased from Heowns Co. (Tianjin, China). Ferric chloride hexahydrate ($FeCl_3 \cdot 6H_2O$), piperazine (PIP, 99%), trimesoyl chloride (TMC, 98%), and other dyes including evans blue (EB), methyl blue (MB), congo red (CR), acid fuchsin (AF), orange G (OG) and methyl red (MR) were purchased from Aladdin Industrial Co. (Shanghai, China). *n*-heptane (99%), sodium sulfate (Na_2SO_4), magnesium sulfate ($MgSO_4$), magnesium chloride ($MgCl_2$), sodium chloride (NaCl) and other chemicals were purchased from Kermel Chemical Reagent Co. (Tianjin, China). Deionized (DI) water used in experiments was manufactured by a Ulupure DI system.

Coordination-driven self-assembly of phytate-coordinated substrates

Phytate-coordinated substrate (PCS) was fabricated by the assembly between phytate and Fe^{3+}. Firstly, PAN membranes were soaked in 25.5 mL phytate solution with varying amounts (113 μL, 225 μL and 450 μL) for 10 min. Subsequently, 4.5 mL $FeCl_3 \cdot 6H_2O$ solution with Fe^{3+} content of 0.1 mol L^{-1} was poured into a phytate solution to trigger the assembly for 10 min. After that, the generated PCS was taken out and transferred into an oven for thermal treatment at 60 °C for 10 min. Finally, PCS was rinsed with DI water (pH=6.0±0.2) for 10 min to remove most residual weakly bound PA molecules and metal ions and stored in DI water before use.

Electrostatic screening of phytate-coordinated substrates

NaCl was introduced as the electrolyte to impact the amine monomer's adsorption. Consequently, PCS was soaked in a 20 mL aqueous solution consisting of PIP (17.5 mg) and NaCl (100 mg) for 10 min before the interfacial polymerization.

Interfacial polymerization of polyamide membranes

To obtain polyamide membranes, the PCS was immersed in a 20 mL aqueous solution of PIP (17.5 mg) for 10 min and dried in the air to remove the residual solution. Afterward, PCS was placed in 20 mL *n*-heptane solution of TMC (13.4 mg) for 15 s to generate a polyamide layer and then taken out for curing at 60 °C for 10 min in an oven.

Characterization

The surface and cross-section morphologies of prepared membranes were observed by field emission scanning electron microscopy (FESEM, Nanosem 430, Japan). More sophisticated morphologies were captured by atomic force microscopy (AFM, Dimension icon, Germany) to characterize membranes' surface roughness and thickness, and the underwater atomic-scale interaction between PIP and the substrate surface. The chemical structure of prepared membranes was detected by Fourier transform infrared spectroscopy (FT-IR/ATR, Nicolet 560, USA), X-ray photoelectron spectroscopy (XPS, ESCALAB Xi+, UK) and EDX spectrum was taken from a Bruker XFlash6|60 energy dispersive X-Ray spectroscopy. To detect the surface wettability of prepared membranes, a contact angle goniometer (JC2000D2, China) was utilized to measure water contact angles. To investigate the charge properties of prepared membranes, an electrokinetic analyzer (Anton Paar KG, Austria) was employed to measure

the surface zeta potential of membranes. The porosity of the PCS was measured by Brunauer–Emmett–Teller (BET, Quantachrome, Autosorbe-1C-VP, America).

Monomer storage capacity measurements of substrates

Substrates were placed in 20 mL aqueous solution of PIP (17.5 mg) for 10 min and dried in the air to remove the residual solution. Afterward, PCS was immersed in 20 mL *n*-heptane. In the meantime, a 1.5 mL solution would be extracted in a different time to be used to detect the concentration of PIP by UV-vis spectrophotometer (Hitachi UV-2800, Japan) and then poured back.

Monomer diffusion rate measurements of substrates

Relative monomer diffusion rate (D_r, h^{-1}) on different substrates was calculated by the following equation:

$$D_r = \frac{S_{PIP\text{-}O} V_O}{A C_{PIP}} \quad (3)$$

where S is the changing rate of PIP (mmol L^{-1} h^{-1}), V_O is the volume of the organic phase (20 mL), A is the surface area of the substrate (15.9 cm^2) and C_{PIP} is the PIP storage capacity (mmol m^{-2}) of the substrate. This calculation referred to the methanol diffusion rate across methanol fuel cell membranes as previously reported(He et al., 2016).

Calculation of surface charge density

The surface charge density (σ, mC m^{-2}) of the substrate was calculated by surface zeta potential according to the Gouy-Chapman equation (Bowen and Cao, 1998) as follows:

$$\sigma = -\varepsilon\kappa\xi \frac{\sinh\left(\frac{F\xi}{2RT}\right)}{\frac{F\xi}{2RT}} \quad (4)$$

where $\kappa^{-1} = \left(\frac{\varepsilon RT}{2F^2C}\right)^{\frac{1}{2}}$ is Debye length (nm), ξ is surface zeta potential (mV), R is gas constant (8.314 J mol^{-1} K^{-1}), F is Faraday constant (96485 C mol^{-1}), T is the absolute temperature (298 K), ε is permittivity (6.933×10^{-10} F m^{-1}).

Calculation of electrostatic interaction energy

The electrostatic interaction energy (ΔE_{EL}, J) was calculated by the following equation in the XDLVO theory(Liu and Zhao, 2005):

$$\Delta E_{EL}(d) = \varepsilon\pi r\left\{(\varphi_1+\varphi_2)^2 \ln\left[1+exp(-\kappa d)\right]+ (\varphi_1-\varphi_2)^2 \ln\left[1-exp(-\kappa d)\right]\right\} \quad (5)$$

where r (nm) is the radius of amine monomer, d (nm) is the distance between the two interacting bodies, κ is the Debye-Huckel parameter (κ^{-1}=1.1 nm) and ε is the electrical

permittivity of the medium or solution (6.95×10^{-10} C^2 J^{-1} m^{-1} for water), φ (mV) is the zeta potential. The surface potential of PIP was calculated according to Coulomb's law as follows:

$$\psi_S = \frac{q}{4\pi er} \tag{6}$$

where Ψ_s (mV) is the surface potential of hydrated ion, q is the charge of protonated PIP, e is dielectric constant, and r (nm) is the radius of the PIP molecule.

Atomic force detecting technology (AFDT) for monomer-substrate interaction

The underwater atomic-scale force between the PIP monomer and the substrate surface was measured with silicon nitride tips (SNL-10, Sharpened, 4 levers 0.0−0.35 N m^{-1}, Au Reflex coating, Bruker) from AFM under contact mode. The air-dried substrate sample was cut into pieces (3 cm× 3 cm) and stick onto quartz slides. 200 uL of PIP aqueous solutions with and without NaCl (5000 ppm) were dropped onto the substrate surface to provide a solid-liquid interface. The SNL-10 tip was immersed in the PIP solution during the whole experiment.

Separation performance measurements

The separation performance of membranes was evaluated using a crossflow filtration apparatus. The membranes were firstly compacted at 2.5 bar for 0.5 hours before measurement at 25±0.5 °C. Sequently the filtration performance was measured at 2.0 bar. The salt rejection was also measured by rejecting different salt solutions (NaCl, Na_2SO_4, $MgSO_4$, $MgCl_2$) and different concentrations (1000, 2000, 3000,4000 and 5000 ppm). The permeance (J, L m^{-2} h^{-1} bar^{-1}), salt rejection ratio (R, %) and Cl^-/SO_4^{2-} selectivity (α) were calculated by the following equations:

$$J = \frac{V}{A\Delta t\Delta P} \tag{7}$$

$$R = \frac{C_f - C_p}{C_f} \times 100\% \tag{8}$$

$$\alpha\left(Cl^- / SO_4^{2-}\right) = \frac{P_{NaCl}}{P_{Na_2SO_4}} \times 100\% = \frac{1 - R_{NaCl}}{1 - R_{Na_2SO_4}} \times 100\% \tag{9}$$

where V (L) is the permeating volume, A (m^2) is the effective membrane area, Δt (h) is the permeating time, ΔP (bar) is the driving pressure, C_p (ppm) and C_f (ppm) are the solute concentration in permeate and feed solutions, respectively while P_{NaCl} and P_{Na2SO4} are permeation ratio of NaCl and Na_2SO_4. Solute concentrations of single salt solution were determined by the conductivity of the solution and detected by an electrical conductivity meter (Leichi, DDS-11A, China). Ion concentrations of mixed salts solution were measured by ion chromatography (Thermo Fisher, USA).

Dye rejection measurements

Dye concentration was determined by a UV-vis spectrophotometer and the 10 ppm dye curves were measured as references for accurate dye rejection. Dye rejection was calculated by the following equation:

$$R = \frac{C_f - C_p}{C_f} \times 100\% = \frac{10 \times A_{f(10ppm)} - A_p}{10 \times A_{f(10ppm)}} \times 100\% \tag{10}$$

where C_p (ppm) and C_f (ppm) are the solute concentration in permeate and feed solutions, respectively.

Water transport resistance and energy measurements

The water transport resistance (R, bar h m^{-1}) across the membrane and the permeance of the polyamide layer (J_p, L m^{-2} h^{-1} bar^{-1}) was calculated by the following equations:

$$J_{Total} = \frac{1}{R_S + R_P} \tag{11}$$

$$R_S = \frac{1}{J_S} \tag{12}$$

$$R_P = \frac{1}{J_{Total}} - \frac{1}{J_S} \tag{13}$$

$$J_P = \frac{1}{R_P} \tag{14}$$

where J_{Total} (L m^{-2} h^{-1} bar^{-1}) and J_S (L m^{-2} h^{-1} bar^{-1}) is the permeance of membrane and substrate, respectively.

The activation energy (E_a, kJ mol^{-1}) for water transport across the membrane was evaluated by measuring water permeance of membranes under varied temperature with the following Arrhenius equation,

$$J = Ae^{(-E_a/RT)} \tag{15}$$

where J is the solvent permeance (L m^{-2} h^{-1} bar^{-1}), A is the pre-exponential factor (L m^{-2} h^{-1} bar^{-1}), E_a is the activation energy associated with the permeation process (kJ mol^{-1}), R is the gas constant (kJ mol^{-1} K^{-1}), and T is the absolute temperature (K). The E_a can be evaluated by taking the logarithm of both sides of Arrhenius equation and using R (8.314×10^{-3} kJ mol^{-1} K^{-1}).

Ionic diffusion measurements

Hydrated ions' diffusion rate was detected by a concentration gradient-driven ion permeation experiment based on an H-shape diffusion cell (**Figure S28**). All salts were adopted with the same concentration (0.1 mol L^{-1}) on the brine side, and the conductivity of water side was measured every 30 minutes to investigate salts concentrations permeating across the membrane.

4. Supplemental References

Bai, L., Liu, Y., Ding, A., Ren, N., Li, G., and Liang, H. (2019). Fabrication and characterization of thin-film composite (TFC) nanofiltration membranes incorporated with cellulose nanocrystals (CNCs) for enhanced desalination performance and dye removal. Chemical Engineering Journal *358*, 1519-1528.

Bowen, W.R., and Cao, X.W. (1998). Electrokinetic effects in membrane pores and the determination of zeta-potential. Journal of Membrane Science *140*, 267-273.

Gao, S., Zhu, Y., Gong, Y., Wang, Z., Fang, W., and Jin, J. (2019). Ultrathin Polyamide Nanofiltration Membrane Fabricated on Brush-Painted Single-Walled Carbon Nanotube Network Support for Ion Sieving. Acs Nano *13*, 5278-5290.

He, G., He, X., Wang, X., Chang, C., Zhao, J., Li, Z., Wuab, H., and Jiang, Z. (2016). A highly proton-conducting, methanol-blocking Nafion composite membrane enabled by surface-coating crosslinked sulfonated graphene oxide. Chemical Communications *52*, 2173-2176.

Ji, Y.-L., An, Q.-F., Weng, X.-D., Hung, W.-S., Lee, K.-R., and Gao, C.-J. (2018). Microstructure and performance of zwitterionic polymeric nanoparticle/polyamide thin-film nanocomposite membranes for salts/organics separation. Journal of Membrane Science *548*, 559-571.

Jiang, C., Tian, L., Zhai, Z., Shen, Y., Dong, W., He, M., Hou, Y., and Niu, Q.J. (2019). Thin-film composite membranes with aqueous template-induced surface nanostructures for enhanced nanofiltration. Journal of Membrane Science *589*, 117244.

Liu, Y., and Zhao, Q. (2005). Influence of surface energy of modified surfaces on bacterial adhesion. Biophysical Chemistry *117*, 39-45.

Pan, Y., Xu, R., Lu, Z., Yu, S., Liu, M., and Gao, C. (2017). Enhanced both perm-selectivity and fouling resistance of poly(piperazine-amide) nanofiltration membrane by incorporating sericin as a co-reactant of aqueous phase. J Membrane Sci *523*, 282-290.

Shi, Q., Ni, L., Zhang, Y., Feng, X., Chang, Q., and Meng, J. (2017). Poly(p-phenylene terephthamide) embedded in a polysulfone as the substrate for improving compaction resistance and adhesion of a thin film composite polyamide membrane. J Mater Chem A *5*, 13610-13624.

Tan, Z., Chen, S., Peng, X., Zhang, L., and Gao, C. (2018). Polyamide membranes with nanoscale Turing structures for water purification. Science *360*, 518-521.

Wang, Z., Wang, Z., Lin, S., Jin, H., Gao, S., Zhu, Y., and Jin, J. (2018). Nanoparticle-templated nanofiltration membranes for ultrahigh performance desalination. Nature Communications *9*, 2004.

Wu, M.-B., Lv, Y., Yang, H.-C., Liu, L.-F., Zhang, X., and Xu, Z.-K. (2016). Thin film composite membranes combining carbon nanotube intermediate layer and microfiltration support for high nanofiltration performances. J Membrane Sci *515*, 238-244.

Wu, M., Ma, T., Su, Y., Wu, H., You, X., Jiang, Z., and Kasher, R. (2017). Fabrication of composite nanofiltration membrane by incorporating attapulgite nanorods during interfacial polymerization for high water flux and antifouling property. J Membrane Sci *544*, 79-87.

Wu, M., Yuan, J., Wu, H., Su, Y., Yang, H., You, X., Zhang, R., He, X., Khan, N.A., Kasher, R., *et al.* (2019). Ultrathin nanofiltration membrane with polydopamine-covalent organic

framework interlayer for enhanced permeability and structural stability. Journal of Membrane Science *576*, 131-141.
You, X., Ma, T., Su, Y., Wu, H., Wu, M., Cai, H., Sun, G., and Jiang, Z. (2017). Enhancing the permeation flux and antifouling performance of polyamide nanofiltration membrane by incorporation of PEG-POSS nanoparticles. Journal of Membrane Science *540*, 454-463.
Yuan, J., Wu, M., Wu, H., Liu, Y., You, X., Zhang, R., Su, Y., Yang, H., Shen, J., and Jiang, Z. (2019). Covalent organic framework-modulated interfacial polymerization for ultrathin desalination membranes. Journal of Materials Chemistry A *7*, 25641-25649.
Zhang, Z., Shi, X., Wang, R., Xiao, A., and Wang, Y. (2019). Ultra-permeable polyamide membranes harvested by covalent organic framework nanofiber scaffolds: a two-in-one strategy. Chemical Science *10*, 9077-9083.
Zhu, J., Hou, J., Zhang, R., Yuan, S., Li, J., Tian, M., Wang, P., Zhang, Y., Volodin, A., and Van der Bruggen, B. (2018a). Rapid water transport through controllable, ultrathin polyamide nanofilms for high-performance nanofiltration. Journal of Materials Chemistry A *6*, 15701-15709.
Zhu, J., Qin, L., Uliana, A., Hou, J., Wang, J., Zhang, Y., Li, X., Yuan, S., Li, J., Tian, M.*, et al.* (2017). Elevated Performance of Thin Film Nanocomposite Membranes Enabled by Modified Hydrophilic MOFs for Nanofiltration. Acs Appl Mater Inter *9*, 1975-1986.
Zhu, J., Yuan, S., Uliana, A., Hou, J., Li, J., Li, X., Tian, M., Chen, Y., Volodin, A., and Van der Bruggen, B. (2018b). High-flux thin film composite membranes for nanofiltration mediated by a rapid co-deposition of polydopamine/piperazine. J Membrane Sci *554*, 97-108.
Zhu, Y., Xie, W., Gao, S., Zhang, F., Zhang, W., Liu, Z., and Jin, J. (2016). Single-Walled Carbon Nanotube Film Supported Nanofiltration Membrane with a Nearly 10 nm Thick Polyamide Selective Layer for High-Flux and High-Rejection Desalination. Small *12*, 5034-5041.
